# Phosphonates and Phosphonate Prodrugs in Medicinal Chemistry: Past Successes and Future Prospects

**DOI:** 10.3389/fchem.2022.889737

**Published:** 2022-05-20

**Authors:** Marcela Krečmerová, Pavel Majer, Rana Rais, Barbara S. Slusher

**Affiliations:** ^1^ Institute of Organic Chemistry and Biochemistry, Academy of Sciences of the Czech Republic (ASCR), Prague, Czechia; ^2^ Departments of Neurology, Pharmacology and Molecular Sciences, Johns Hopkins Drug Discovery, Baltimore, MD, United States; ^3^ Departments of Neurology, Pharmacology and Molecular Sciences, Psychiatry and Behavioral Sciences, Neuroscience, Medicine, Oncology, Johns Hopkins Drug Discovery, Baltimore, MD, United States

**Keywords:** acyclic nucleoside phosphonates, antivirals, prodrugs, protides, GCPII, prostate-specific membrane antigen, FOLH1, 2-PMPA

## Abstract

Compounds with a phosphonate group, i.e., –P(O)(OH)_2_ group attached directly to the molecule *via* a P-C bond serve as suitable non-hydrolyzable phosphate mimics in various biomedical applications. In principle, they often inhibit enzymes utilizing various phosphates as substrates. In this review we focus mainly on biologically active phosphonates that originated from our institute (Institute of Organic Chemistry and Biochemistry in Prague); i.e., acyclic nucleoside phosphonates (ANPs, e.g., adefovir, tenofovir, and cidofovir) and derivatives of non-nucleoside phosphonates such as 2-(phosphonomethyl) pentanedioic acid (2-PMPA). Principal strategies of their syntheses and modifications to prodrugs is reported. Besides clinically used ANP antivirals, a special attention is paid to new biologically active molecules with respect to emerging infections and arising resistance of many pathogens against standard treatments. These new structures include 2,4-diamino-6-[2-(phosphonomethoxy)ethoxy]pyrimidines or so-called “open-ring” derivatives, acyclic nucleoside phosphonates with 5-azacytosine as a base moiety, side-chain fluorinated ANPs, aza/deazapurine ANPs. When transformed into an appropriate prodrug by derivatizing their charged functionalities, all these compounds show promising potential to become drug candidates for the treatment of viral infections. ANP prodrugs with suitable pharmacokinetics include amino acid phosphoramidates, pivaloyloxymethyl (POM) and isopropoxycarbonyloxymethyl (POC) esters, alkyl and alkoxyalkyl esters, salicylic esters, (methyl-2-oxo-1,3-dioxol-4-yl) methyl (ODOL) esters and peptidomimetic prodrugs. We also focus on the story of cytostatics related to 9-[2-(phosphonomethoxy)ethyl]guanine and its prodrugs which eventually led to development of the veterinary drug rabacfosadine. Various new ANP structures are also currently investigated as antiparasitics, especially antimalarial agents e.g., guanine and hypoxanthine derivatives with 2-(phosphonoethoxy)ethyl moiety, their thia-analogues and N-branched derivatives. In addition to ANPs and their analogs, we also describe prodrugs of 2-(phosphonomethyl)pentanedioic acid (2-PMPA), a potent inhibitor of the enzyme glutamate carboxypeptidase II (GCPII), also known as prostate-specific membrane antigen (PSMA). Glutamate carboxypeptidase II inhibitors, including 2-PMPA have been found efficacious in various preclinical models of neurological disorders which are caused by glutamatergic excitotoxicity. Unfortunately its highly polar character and hence low bioavailability severely limits its potential for clinical use. To overcome this problem, various prodrug strategies have been used to mask carboxylates and/or phosphonate functionalities with pivaloyloxymethyl, POC, ODOL and alkyl esters. Chemistry and biological characterization led to identification of prodrugs with 44–80 fold greater oral bioavailability (tetra-ODOL-2-PMPA).

## 1 Introduction

Phosphonates are compounds bearing a phosphonate (P(O)(OH)_2_) group attached to the molecule *via* a P-C bond. They serve as non-hydrolyzable phosphate mimics in various biomedical applications. Usually, they inhibit enzymes utilizing phosphates as substrates. Some phosphonates occur in nature as natural phosphonate antibiotics (e.g., fosfomycin). Some important synthetic phosphonate-based drugs include bisphosphonates (for the treatment of osteoporosis) and the antivirals foscarnet and besifovir, and perzintofel (for the treatment of stroke). In this review we focus on two groups of biologically active phosphonates. The first originated from IOCB in Prague (acyclic nucleoside phosphonates; ANPs) and the second originated from a collaboration between IOCB and the Johns Hopkins Drug Discovery team [prodrugs of 2-(phosphonomethyl) pentanedioic acid (2-PMPA)].

## 2 Acyclic Nucleoside Phosphonates (ANPs)

The common structural attribute of ANPs is a nucleobase attached to an aliphatic side chain and containing a phosphonomethyl residue. A methylene bridge between the phosphate moiety and the rest of the molecule excludes enzymatic dephosphorylation. The absence of the glycosidic bond increases resistance to chemical and biological degradation. Flexibility of acyclic chains enables these compounds to adopt conformations suitable for interaction with active sites of enzymes. Their biological activities are mostly antiviral but also cytostatic, immunomodulatory and antiparasitic. Their disadvantage are unfavourable pharmacological properties due to the presence of polar phosphonic acid functionality. ANPs are mostly impermeant to the cellular membrane and in addition, their absorption by gastrointestinal tract is limited which is disqualifying for oral application. To achieve oral bioavailability and intracellular delivery, their transformation to prodrugs is highly advisable.

More than three decades of systematic investigations of ANPs in our institute resulted in hundreds of their structural variations. Three of them are commercially available pharmaceuticals approved for the treatment of viral infections (cidofovir, adefovir and tenofovir). The mentioned compounds represent three different types of ANPs: HPMP derivatives, i.e., (S)-[3-hydroxy-2-(phosphonomethoxy)propyl] derivatives (e.g., (S)-HPMPC, cidofovir), PME derivatives, i.e., 2-(phosphonomethoxy)ethyl derivatives (e.g., PMEA, adefovir) and PMP derivatives, i.e., (*R*)-2-(phosphonomethoxy)propyl derivatives [e.g., (*R*)-PMPA, tenofovir]. This large topic became a subject of many reviews, especially by Antonín Holý and Erik De Clercq (Holý, 2003; De Clercq and Holý, 2005; De Clercq, 2007; De Clercq, 2011; De Clercq, 2013).

Syntheses of acyclic nucleoside phosphonates (ANPs) are based on several alternative approaches: 1) preparation of *N*-(hydroxyalkyl) derivatives of purine or pyrimidine bases followed by introduction of the phosphonomethyl residue, 2) alkylation of the nucleobase with an appropriate synthetic precursor, usually dialkyl ester of phosphonomethoxyalkyl halide or tosylate, 3) ring-closure reactions of some aminoalkylphosphonates forming appropriate heterocyclic base moieties (preparation of aza and deazapurine ANPs) and 4) transformation of reactive functional groups at the side chain or in the heterocyclic base in a previously prepared phosphonate (Holý, 2003).

### 2.1 Acyclic Nucleoside Phosphonate Drugs and Drug Candidates. Synthesis, Clinical Applications and Current Topics in Their Research

#### 2.1.1 Synthesis of (*S*)-HPMP Derivatives

**FIGURE 1:**
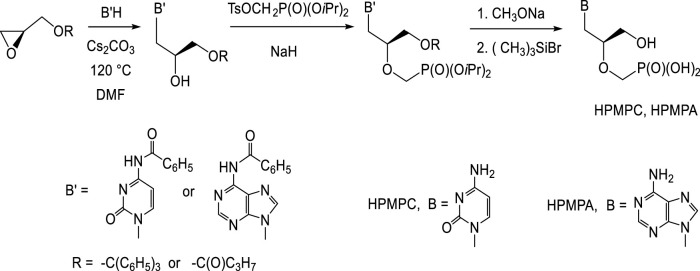
Synthesis of (*S*)-[3-hydroxy-2-(phosphonomethoxy)propyl] derivatives.

Preparation of (*S*)-HPMP derivatives (HPMPC, HPMPA, HPMPDAP, HPMP-5azaC) mostly utilizes the first approach: base catalysed nucleophilic opening of the oxirane ring in (*S*)-2-(trityloxymethyl)oxirane or (*S*)-glycidol butyrate with appropriate nucleobase (Figure 1). Thus created 3-O-substituted (*S*)-2,3-dihydroxypropyl derivatives are then treated with diisopropyl tosyloxymethanephosphonate and finally deprotected. Preparation of diisopropyl tosyloxymethanephosphonate consists in the treatment of diisopropyl phosphite with paraformaldehyde and triethylamine followed by tosylation (Holý, 1993). The alternative way is introduction of phosphonomethyl ether group using diisopropyl bromomethylphosphonate (Göbel et al., 1992; Holý, 2005; Oh and Hong, 2008).

#### 2.1.2 Cidofovir and its Prodrugs

Cidofovir has unique activity against practically all DNA viruses. Intracellularly, it is converted to cidofovir diphosphate which suppresses virus replication selectively by competitive inhibition of viral DNA polymerase. Incorporation of the drug disrupts further chain elongation. Under the brand name Vistide™, cidofovir was approved for the treatment of cytomegalovirus retinitis in AIDS patients (Plosker and Noble, 1999; Drugs.com, 2021). Off label it is used for the treatment of severe cases of papillomatoses (Gi et al., 2012), progressive multifocal leukoencephalopathy (Andrei et al., 2015), adenovirus infections (Neofytos et al., 2007) and poxvirus infections, e.g., recalcitrant molluscum contagiosum and orf in immunocompromised patients (Cundy, 1999; Andrei and Snoeck, 2010). In justified cases, it was exceptionally applied also to immunocompetent pediatric patients with very complicated adeno-, polyoma- or cytomegalovirus infections (Neant et al., 2018; Alcamo et al., 2020).

Application of cidofovir is possible only intravenously and its bioavailability is very low. More than 90% of its intravenous dose is excluded unchanged by kidney. Nephrotoxicity of the cidofovir can be partially overcome by the pre-treatment with the nephroprotective drug probenecid (Lacy et al., 1998; Wolf et al., 2003).

**FIGURE 2:**
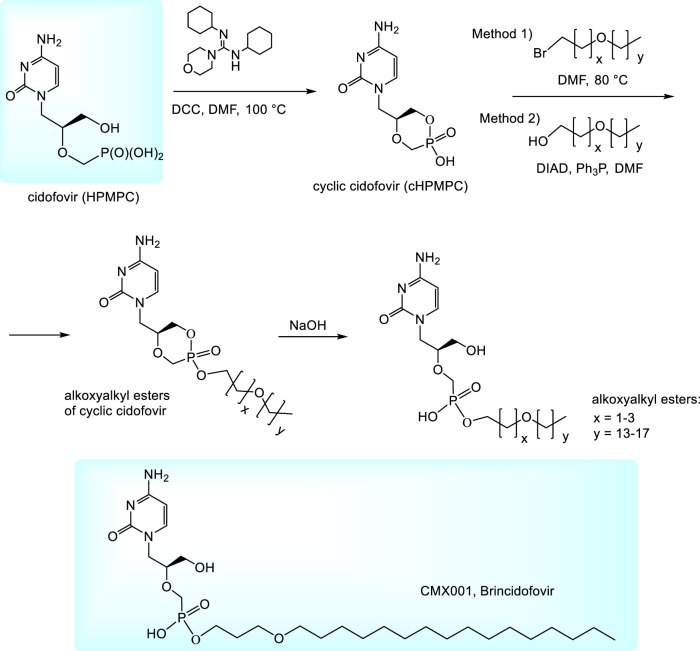
Synthesis of alkoxyalkyl ester prodrugs of cidofovir.

To improve pharmacokinetic properties of cidofovir, various prodrugs are being investigated. So far the most important prodrugs are alkoxyalkyl esters (Kern et al., 2002; Beadle et al., 2002; Ruiz, 2011). They can be prepared by alkylation of *N,N,-*dicyclohexyl-4-morpholinocarboxamidinium salt of cyclic cidofovir (cHPMPC) with appropriate alkoxyalkyl bromides (Method 1, Figure 2) (Beadle et al., 2002; Kern et al., 2002) or condensation of cHPMPC with alkoxyalcohols under Mitsunobu conditions (Method 2) (Ruiz, 2011). Thus formed esters of cyclic HPMPC are subsequently cleaved to corresponding monoesters by the heating with aqueous sodium hydroxide. The method is applicable also for various other phosphonates (not only in the HPMP series) (Hostetler, 2009). Hexadecyloxypropyl (HDP) ester of cidofovir (Brincidofovir, CMX001) was being developed by Chimerix, Inc. as a drug against cytomegalovirus and adenovirus infections in transplant recipients. Oral brincidofovir was investigated in the Phase III of clinical trials for CMV prophylaxis in stem cell transplants (Marty et al., 2019) but the trials failed due to the toxicity in gastrointestinal tract. Also two other Phase III trials for its use in preventing infection after kidney transplantation and some trials targeted to adenovirus were not successful (Marty et al., 2019; Clinical.trials.gov.; Brincidofovir, 2021). It is speculated that the compound tolerability may be improved by a change in the drug formulation, e.g., by intravenous application. The present plans with brincidofovir are targeted mostly to poxviruses, especially variola virus for the case of a smallpox outbreak. Finally, in June 2021, brincidofovir was approved by FDA as a drug for the treatment of smallpox and is marketed under the brand name Tembexa (FDA, 2021). While the smallpox is eradicated in nature, there is still possibility of variola virus abuse as a biological weapon or its accidental release (FDA, 2021). During Ebola outbreak in 2014 Chimerix also received an FDA approval for investigational applications of brincidofovir for the treatment of Ebola virus disease in patients and approval for the Phase II clinical trials. The trials were subsequently discontinued because of a lack of suitable subjects. Anyway, they highlighted the need to establish better practices for preclinical *in vitro* and animal screening of therapeutics for potentially emerging epidemic infectious diseases prior to their use in patients (Dunning et al., 2016).

#### 2.1.3 Synthesis of Purine *N*-2-(Phosphonomethoxy)ethyl (PME) Derivatives

Synthesis of acyclic nucleoside phosphonates of the PME series consists in the base catalysed condensation of a purine or pyrimidine base with a relevant synthetic precursor, i.e., compound containing the whole aliphatic part including a phosphonomethyl arrangement and an appropriate leaving group (mostly tosyl or halogene).

**FIGURE 3:**
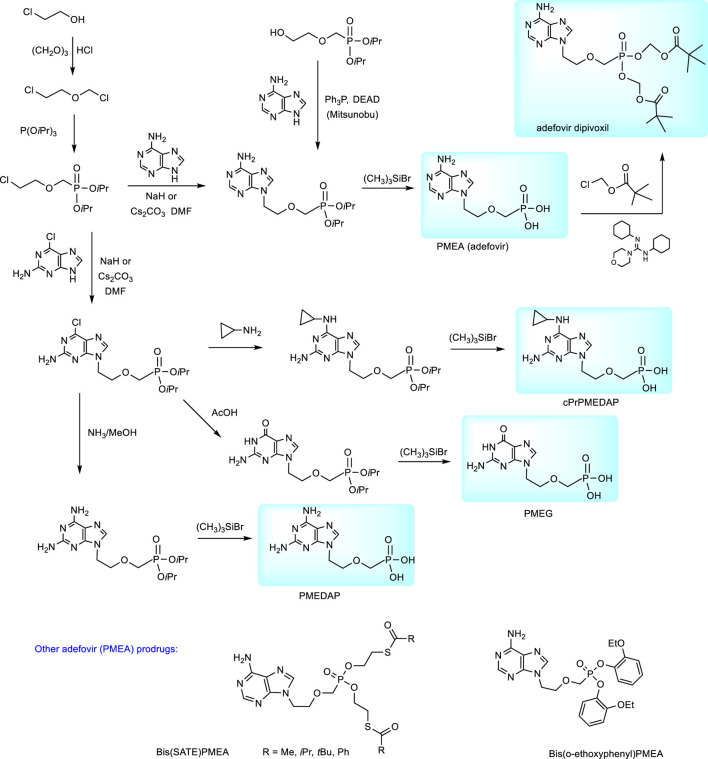
Synthesis of purine *N*-2-(phosphonomethhoxy)ethyl (PME) derivatives.

*N*-2-(Phosphonomethoxy)ethyl derivatives are synthesized from dialkyl (diethyl or diisopropyl) 2-(chloroethoxy)methylphosphonates. These precursors are accessible by the treatment of trialkyl phosphites with 2-(chloroethoxy)methyl chloride (Holý et al., 1999); Holý et al., 1989) The synthesis of PMEA (adefovir) according to this procedure is described in Figure 3. Analogous procedures were applied also to the preparation of the cytostatic guanine derivative PMEG and the series of N^6^-substituted PMEDAP, i.e., 9-[2-(phosphonomethoxy)ethyl]-2,6-diaminopurine including 9-[(2-phosphonomethoxy)ethyl]-N^6^-cyclopropyl-2,6-diaminopurine (cPrPMEDAP, the compound which amidate prodrug is used as a veterinary drug rabacfosadine). In these cases condensation of diisopropyl 2-(chloroethoxymethyl)phosphonate is performed with 2-amino-6-chloropurine and the resulting product was further transformed according to Figure 3 (Holý et al., 2001).

**FIGURE 4:**
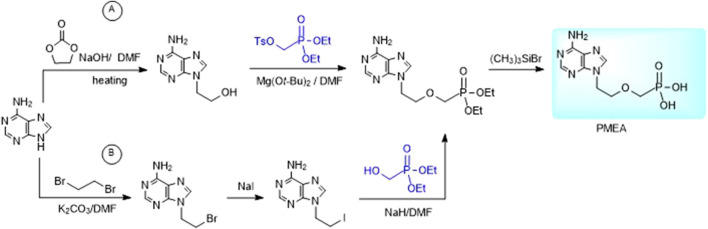
Alternative syntheses of PMEA.

Alternative way to 2-(phosphonomethoxy)ethyl derivatives is the reaction of purine bases with dialkyl 2-hydroxyethylphosphonate under Mitsunobu conditions (Chen et al., 1996). Other methods utilize reaction of dialkyl tosyloxymethylphosphonate with an appropriate 9-(2-hydroxyethyl)purine derivative (Figure 4A) or reaction of an analogous purine 2-iodoethyl derivative with diethyl hydroxymethylphosphonate (Figure 4B on the example of adefovir). The advantage of the latter one is commercial availability of diethyl hydroxymethylphosphonate (Jones et al., 2019).

#### 2.1.4 Adefovir Dipivoxil

Adefovir is active against retroviruses and some types of DNA viruses (Naesens et al., 1994; Holý, 2003; De Clercq and Holý, 2005; De Clercq, 2007). Its activity against HIV and hepatitis B virus consists in the inhibitory effect towards reverse transcriptase.

Synthesis of adefovir prodrug (adefovir dipivoxil, Bis(POM)-PMEA) proceeds from adefovir by the action of chloromethyl pivalate (Figure 3) (Starrett et al., 1992).

Adefovir in the form of bis(pivaloyloxymethyl) ester was being developed originally as anti-HIV agent but the trials were finally discontinued due to the nephrotoxicity at the required daily therapeutic dose 120 mg (Kahn et al., 1999). On the other hand, the efficacy for HBV was found higher when the therapeutic effect was achieved with a ten times lower dose of 10 mg (Izzedine et al., 2004; Fontaine et al., 2005). It enabled development of the compound for the treatment of chronic hepatitis B in adults, especially for those with lamivudine resistance. In 2002, adefovir dipivoxil was approved by FDA under the trade name Hepsera^TM^. Clinical practice shows that sometimes a higher dose of the drug would have been more efficacious but of course, it is ruled out due to the renal safety (Izzedine et al., 2004; Hézode et al., 2007). In rare cases, it is also reported that prolong treatment with adefovir even in a low dose of 10 mg can cause adefovir-induced osteomalacia, a metabolic bone disease that leads to softening of the bones. It is caused by hypophosphatemia as a result of renal tubular dysfunction (Kim et al., 2013; Park et al., 2018). Clinical use of adefovir dipivoxil started to grow down since the more effective tenofovir disoproxil fumarate and later on, tenofovir alafenamide were approved for HBV (2008 and 2016).

#### 2.1.5 Alternative Prodrugs of Adefovir

Alternative prodrug approaches for adefovir to improve adefovir pharmacokinetics were also investigated, e.g., bis(S-acyl-2-thioethyl esters (SATE-esters) (Benzaria et al., 1996) or substitutes aryl esters, e.g., bis(o-ethoxyphenyl) ester (Figure 3) (Shaw et al., 1997). The bis(tBu-SATE)PMEA has 50-times increased stability in human plasma compared to bis(POM)-PMEA and improved stability in human gastric fluid (Benzaria et al., 1996).

**FIGURE 5:**
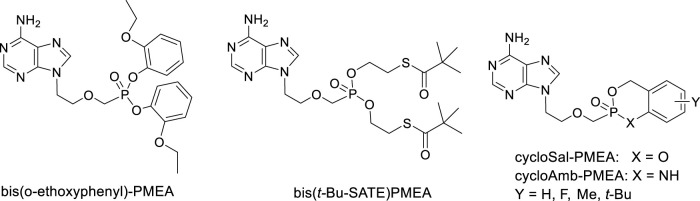
Alternative prodrugs of PMEA.

Extensive research was paid also to cyclosaligenyl (cycloSal) phosphonates, i.e., cyclic esters with variously substituted salicyl alcohol and cycloAmb phosphonates (cyclic ester amidates with 2-aminobenzyl alcohol). Investigation of anti-HIV-1 and anti-HIV-2 activity of various cycloSal-PMEA on cell cultures revealed increased antiviral activity compared to the parent PMEA and in parallel, decreased toxicity and decreased stability compared to other types of prodrugs [e.g., SATE or bis(amidates)]. cycloAmb-derivatives had generally increased stability and lower antiviral activity in comparison with cycloSal-PMEAs (Meier et al., 2005). (Figure 5)

**FIGURE 6:**
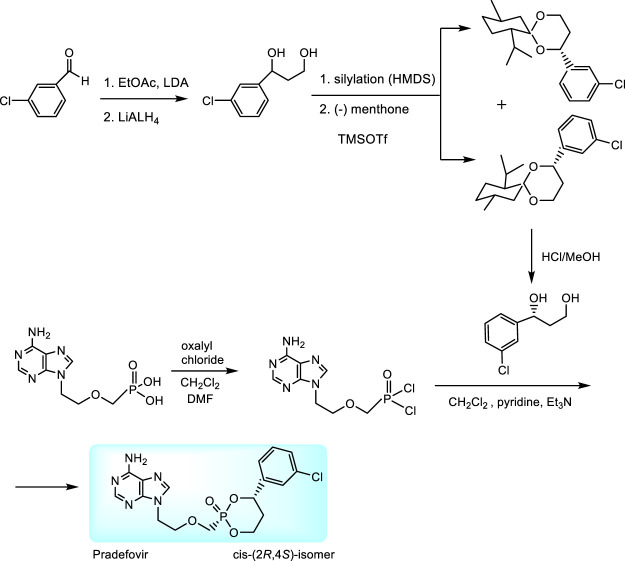
Synthesis of pradefovir.

An original prodrug technology are HepDirect^TM^ prodrugs. They were developed by Ligand Pharmaceuticals, Inc. with the aim to target adefovir directly to the liver due to specific activation by enzymes overexpressed in the liver tissue (Erion et al., 2004; Tillmann, 2007; Reddy et al., 2008). HepDirect prodrugs are cyclic 1-aryl-1,3-propanyl esters which are susceptible to oxidative cleavage of the ring by a cytochrome P450 isozyme (CYP3A4) (Erion et al., 2004). Chemical synthesis consists in condensation of adefovir with 1-(3-chlorophenyl)-1,3-propanediol. While only *cis*-(2*R*,4*S*)-isomer was found as appropriate prodrug, the synthesis includes stereoselective resolution of racemic intermediate 1-(3-chlorophenyl)-1,3-dihydroxypropane through diastereomeric menthone adducts (Figure 6) (Reddy et al., 2008).

Pradefovir was advanced to Phase II of clinical trials where a 12-fold improvement in the liver/kidney ratio over adefovir dipivoxil has been proven. Further investigation in United States was discontinued when the more effective tenofovir disoproxil fumarate became available. Nevertheless, pradefovir is still in clinical development for Hepatitis B in China. Promising results of their Phase 1b study have been recently published (Zhang et al., 2020).

**FIGURE 7:**
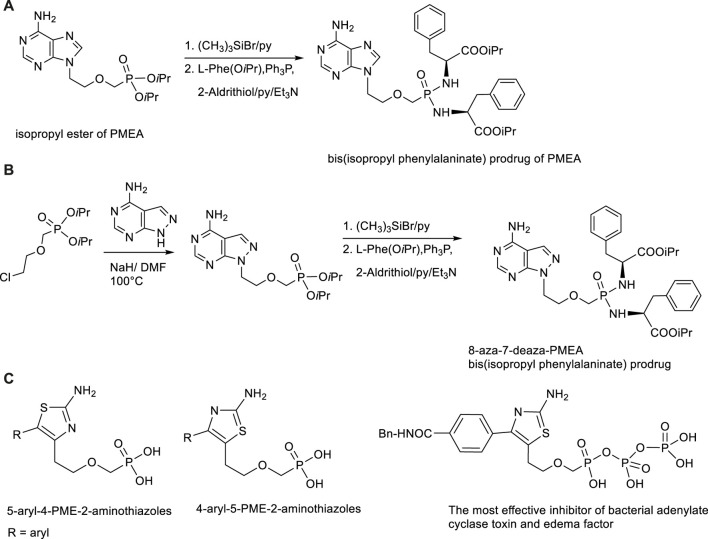
Synthesis and structures of inhibitors of bacterial adenlylate cyclases. **(A)** Synthesis of bis(amidate) prodrug of adefovir. **(B)** Synthesis of bis(amidate) prodrug of 8-aza-7-deaza analogue of adefovir. **(C)** Structures of 5-aryl-4-PME-2-aminothiazoles and 4-aryl-5-PME-2-aminothiazoles.

Besides antiviral effects extensive research was targeted to antibacterial activity of PMEA prodrugs. Studies performed with Bis(POM)PMEA revealed that adefovir upon intracellular conversion into the active metabolite adefovir diphosphate has strong activity against adenylate cyclase toxin (ACT) from *Bordetella* *pertussis*, a causal agent of whooping cough, and both ACT and edema factor (EF) from *Bacillus anthracis*. Various symmetrical amino acid ester based bis(amidates) of PMEA have been developed in our Institute as less toxic and more stable alternatives to bis(POM)PMEA (Česnek et al., 2015; Břehová et al., 2016; Šmídková et al., 2014). The highest activity and optimal pharmacokinetic profile was achieved with bis(isopropyl phenylalanine) amidate promoiety. Besides, also various base modified PMEA analogues (aza/deaza purine derivatives) were synthesized. One of the most promising ACT inhibitors was found 8-aza-7-deazapurine analogue of PMEA in the form of bis(isopropyl phenylalanine) amidate with IC_50_ value16 nM and substantial selectivity over mammalian adenylate cyclases (Česnek et al., 2018). Synthesis of symmetrical bis(amidates) can be performed from phosphonate diisopropyl esters which are deprotected first with bromotrimethylsilane, followed by condensation with appropriate amino acid ester in the presence of 2,2′-dithiopyridine (Aldrithiol). The reaction scheme is outlined in Figure 7. Extraordinary effect to ACT inhibitory potency has replacement of the adenine moiety with another heterocyclic base able to mimic adenine, namely 2-aminothiazole. These compounds are 5-aryl-4-PME-2-aminothiazoles, 4-aryl-5-PME-2-aminothiazoles and their bis(amidate)prodrugs and phosphono diphosphate analogues (Břehová et al., 2021; Česnek at al., 2022). The most potent inhibitor was diphosphate of 4-(4-(benzylcarbamoyl)phenyl-5-PME-2-aminothiazole (Figure 7). It is the most potent ANP-based inhibitor of ACT (IC_50_ = 9 nM) and EF (IC_50_ = 11.6 nM) known to date.

#### 2.1.6 Cytostatic PME Derivatives: PMEG, cPrPMEDAP and Rabacfosadine (GS-9219, VetDC-1101, Tanovea^TM^)

Several years ago, a considerable attention started to be paid to cytostatic 9-[2-(phosphonomethoxy)ethyl] derivatives derived from guanine, 2,6-diaminopurine and N^6^-substituted 2,6-diaminopurine (Figure 3). These compounds work *via* perturbing DNA replication by terminating the growing DNA chain and suppressing the cell growth (at low concentrations) while at higher concentrations they induce apoptotic activity. In cells, they are phosphorylated to diphosphates as active metabolites inhibiting the cell growth due to a potent inhibition of nuclear DNA polymerases. It results in inhibition of DNA synthesis and/or DNA repair (Kramata et al., 1996; Pisarev et al., 1997; Kramata et al., 1998). The most potent compound is PMEG, 9-[(2-phosphonomethoxy)ethyl]guanine; the activities generally increase in the order PMEA < PMEDAP < PMEG. Antitumor effects of PMEG was proven also in animal models in a Sprague–Dawley rat experimental model for T-cell lymphoblastic leukemia/lymphoma (Rose et al., 1990; Holý, 2003) but the utility of PMEG for clinical practice is very limited by its poor cellular permeability and toxicity (Kreider et al., 1990; Rose et al., 1990). A fundamental breakthrough was made by the N^6^-substitution of the 6-amino group in 2,6-diamino-9-[2-(phosphonomethoxy)ethyl]purine (PMEDAP) leading to numerous compounds with very promising activity (Holý et al., 2001). Finally, the cyclopropyl derivative, cPrPMEDAP was selected as the lead compound. Its antiproliferative effects were similar to PMEG *in vitro*, however, *in vivo*, toxicity of cPrPMEDAP was substantially reduced. In cell cultures, cPrPMEDAP is deaminated to PMEG and then converted to the PMEG-diphosphate—it is evident that cPrPMEDAP acts as an intracellular prodrug of PMEG (Compton et al., 1999; Hatse et al., 1999). The enzyme capable of this conversion was identified as N^6^-methyl-AMP/dAMP aminohydrolase (Schinkmanová et al., 2006; Schinkmanová et al., 2008).

**FIGURE 8:**
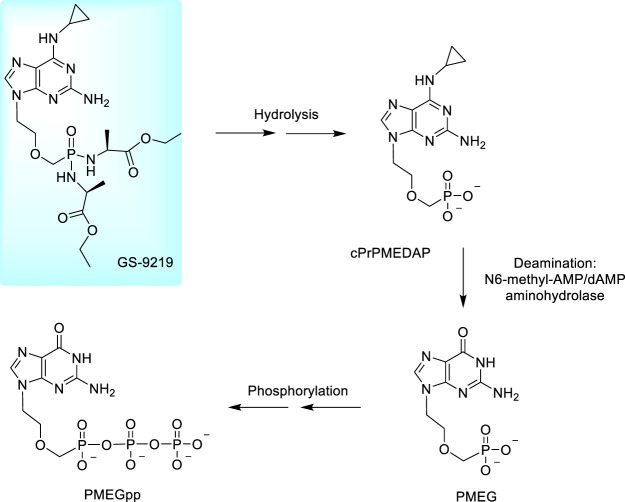
Metabolic conversion of rabacfosadine to PMEG diphosphate.

Nevertheless, cPrPMEDAP, similarly as other ANPs, has a low cellular permeability making its direct clinical use practically impossible and transformation to an appropriate prodrug was necessary. Development of amino acid amidate prodrugs resulted finally in the development of compound GS-9219, the ethyl alaninate prodrug of cPrPMEDAP and concurrently a double prodrug of PMEG. The compound was designed as a cytotoxic agent preferentially targeting lymphoid cells. The compound is prepared from cPrPMEDAP by the action of L-alanine ethyl ester hydrochloride after activation of phosphonic acid residue with Aldrithiol and triphenylphosphine (Cheng et al., 2005; Jansa et al., 2011). The scheme of metabolic conversion of GS-9219 to PMEGpp is outlined in Figure 8. Preclinical trials with dogs with spontaneous non-Hodgkin’s lymphoma proved GS-9219 as generally well tolerated drug with significant antitumor activity warranting its further study in humans (Reiser et al., 2008).

Clinical trials of GS-9219 (Phase I/II) were started in July 2007. The compound was tested in adult patients with non-Hodgkin’s lymphoma, chronic lymphocytic leukemia and multiple myeloma in twenty leading experimental clinics in United States, Russia and Czech Republic. Despite expectations, 3 years later, in October 2010, the study on GS-9219 has been terminated due to the unacceptable safety profile of the compound (Clinical.trials.gov. GS-9219, 2014). Later on, it was licensed to the veterinary drug company VetDC where the compound was being developed as a veterinary drug against canine lymphoma (VDC-1101, rabacfosadine). In April 2017, it has been approved by FDA as a veterinary drug. The drug is currently marketed by Elanco Animal health under the trade name Tanovea^TM^ (Elanco, 2021). The whole story has been recently reviewed by De Clercq (De Clercq, 2018).

#### 2.1.7 *N*-[2**-**(Phosphonomethoxy)Propyl (PMP) Derivatives Tenofovir and its Prodrugs

**FIGURE 9:**
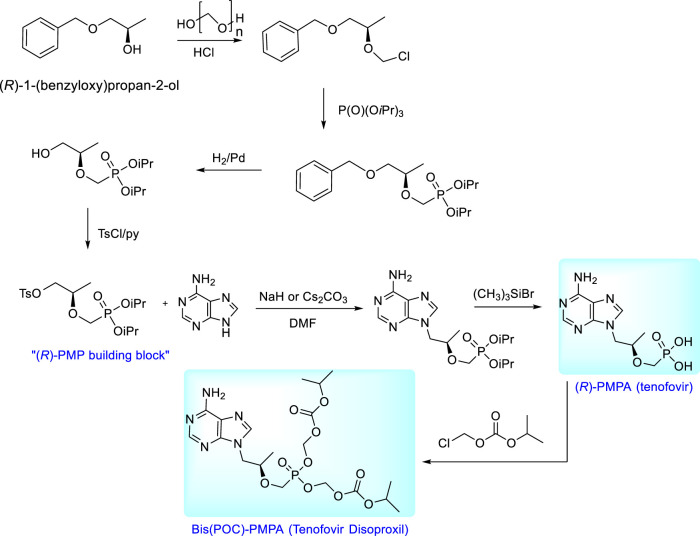
Synthesis of tenofovir and its prodrug, tenofovir disoproxil.

Syntheses of enantiomeric *N*-[2-(phosphonomethoxy)propyl derivatives are based on the condensation of a nucleobase with the appropriate chiral building block. The starting compound for the synthesis of (*R*)-PMPA (tenofovir) is (*R*)-2-[bis(2-propyl)phosphonomethoxy]propyl]-p-toluenesulfonate, i.e., “(*R*)-PMP building block”, the compound prepared easily from (*R*)-1-benzyloxy-2-propanol by multi-step process involving chloromethylation, Arbuzov reaction with triisopropyl phosphite, catalytic hydrogenation and final tosylation (Holý et al., 1995). Condensation of adenine with this precursor followed by deprotection of ester groups gives (*R*)-9-[(2-phosphonomethoxy)propyl]adenine [(*R*)- PMPA, tenofovir]. For clinical utilization this compound is transformed to the neutral prodrug, bis(isopropoxycarbonyloxymethyl) ester [tenofovir disoproxil, Bis(POC)- (*R*)-PMPA] and marketed in the form of fumarate salt under the trade name Viread^TM^. The prodrug is synthesized from (*R*)-PMPA and chloromethyl isopropyl carbonate (Arimilli et al., 1997; Arimilli et al., 1999). The overall scheme of the synthesis of tenofovir and its bis(POC) prodrug is outlined in Figure 9.

Tenofovir is a typical antiretroviral agent acting as a nucleotide reverse transcriptase inhibitor (Srinivas and Fridland, 1998; Suo and Johnson, 1998; Cihlar et al., 2002). In 2001, its bis(isopropoxycarbonyloxymethyl) ester (tenofovir disoproxil fumarate, TDF) was approved for the treatment of HIV infections as one-component drug Viread^TM^ (Srinivas and Fridland, 1998; Lyseng-Williamson, 2005). Current trend in anti-HIV therapy is a combined antiretroviral therapy (cART) consisting in several anti-HIV drugs with different mechanism of action in all-in-one pill. Marketed combinations of TDF are: Truvada (TDF + emtricitabine), Atripla (TDF + emtricitabine + efavirenz), Complera (emtricitabine + rilpivirine) and Stribild (TDF + elvitegravir + cobicistat + emtricitabine) (Gilead, 2022; Deeks and Perry, 2010; De Clercq, E., 2019).

Tenofovir is the most effective, safe and best selling anti-HIV drug. Its special advantages are preventive effects in mother-to-child HIV transmission and relative safety for pregnant women and pediatric patients (Nachega, et al., 2017; Gibb et al., 2012; Siberry et al., 2012; Wang et al., 2013). In 2012, FDA approved Truvada (emtricitabine/tenofovir disoproxil fumarate) as the first drug effective to reduce the risk of HIV transmission to uninfected individuals. Extensive investigations are also carried out in the field of preventive effects of tenofovir containing microbicides. Several clinical studies including daily use of tenofovir vaginal gel or its combination with oral tenofovir are under way in several African countries. Despite an initial enthusiasm from the results of the Caprisa 004 study published in 2010, the following studies (VOICE and FACTS 001) failed due to a failure of the human factor: An adherence in all aspects of the study was too low to confirm effectiveness. The same result was obtained from the FEM-PrEP clinical trial designed to assess prevention of HIV infection with a daily dose of one pill of Truvada (Van Damme et al., 2012; Marrazzo et al., 2015).

Tenofovir has been also investigated as anti-hepatitis B agent and in 2008 approved by FDA for the treatment of chronic hepatitis B in adults and pediatric patients 12 years of age and older. Tenofovir disoproxil is more efficacious than previously approved adefovir dipivoxil, and so far there is no reported resistance (Yang et al., 2015; Tillmann and Samuel et al., 2019; de Fraga et al., 2020). Both ANPs (tenofovir and adefovir) act as chain terminators when their metabolites get incorporated into the viral DNA strands while they undergo replication by polymerases or reverse transcriptase. However, recent discoveries showed also an additional immunomodulatory mechanism. Both ANP drugs, (but not nucleoside analogues), induce interferon (IFN)-λ3 in the gastrointestinal tract. Pretreatment of peripheral blood mononuclear cells from HBV patients with these ANPs inhibited LPS-mediated interleukin (IL)-10 production (Murata et al., 2020).

**FIGURE 10:**
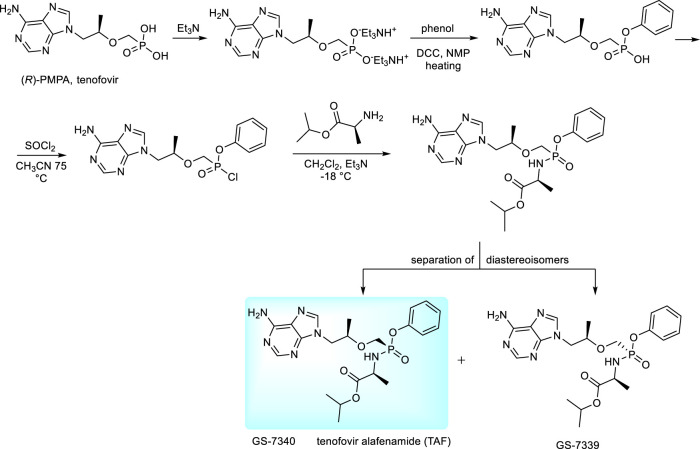
Synthesis of proTides derived from tenofovir.

The new prodrug form of tenofovir developed by Gilead Sciences is tenofovir alafenamide (TAF) (De Clercq, 2016; Lee et al., 2005; Birkus et al., 2017). It is preferentially taken up by the lymphatic tissue and, also by liver cells (Birkus et al., 2017). The compound is a typical representative of “ProTides” - aryloxy amino acid phosphoramidates, the technology developed originally by C. McGuigan. “ProTides” are widely used due to optimal pharmacokinetic properties and relatively easy synthesis (Pradere et al., 2014; Heidel and Dowd, 2019). Synthesis of TAF has been described in many patents, including variations of experimental conditions and separation of diastereoisomers (GS-7340 and GS-7339, Figure 10) (Chapman et al., 2001; Ramanathan, 2013). The large-scale synthesis and process for separation of diastereomers by simulated moving bed chromatography (SMBC) is described in ref. (Chapman et al., 2001). The desired isomer was found GS-7340 with anti-HIV-1 activity data about ten-fold higher than GS-7339. For clinical application, the compound is used in the form of hemifumarate (Liu, 2013). For HBV therapy, it is sold under the brand name Vemlidy. At present, TAF has largely replaced TDF in HIV treatments, primarily due to the significant difference in dosage—only 30 vs. 300 mgs, with lower incidence of adverse side effects as well as greatly increased levels of tenofovir inside the virally infected cells (De Clercq, 2016; Seley-Radtke and Yates, 2018; De Clercq, 2019). TAF is also used in various combinations with other antiretroviral drugs (Genvoya, Odefsey, Descovy, Symtuza).

### 2.2 Current Trends and Future Perspectives

Despite the progress in modern medicine, there will be always need for new biologically active molecules. The reason are emerging infections, arising resistance of many pathogens against standard treatments and increasing number of patients with immunity problems. Many ANPs are active against serious, life threatening infections still lacking effective treatment e.g., cytomegalovirus and polyomavirus infections (in perinatal patients and transplant or other immunocompromised patients), poxvirus infections and specific herpesvirus infections (e.g., human herpesvirus type 6, HHV-6). All these facts substantiate further investigations in the field which are targeted to the following areas:

1 Compounds from Antonin Holy´s legacy—structures with excellent biological activities still waiting for clinical/preclinical investigations
2 Development of new ANP structures
3 New ANP prodrugs with improved pharmacokinetic properties
4 New targets and new applications

#### 2.2.1 “Old Compounds” Still Waiting for Their Opportunity

**FIGURE 11:**
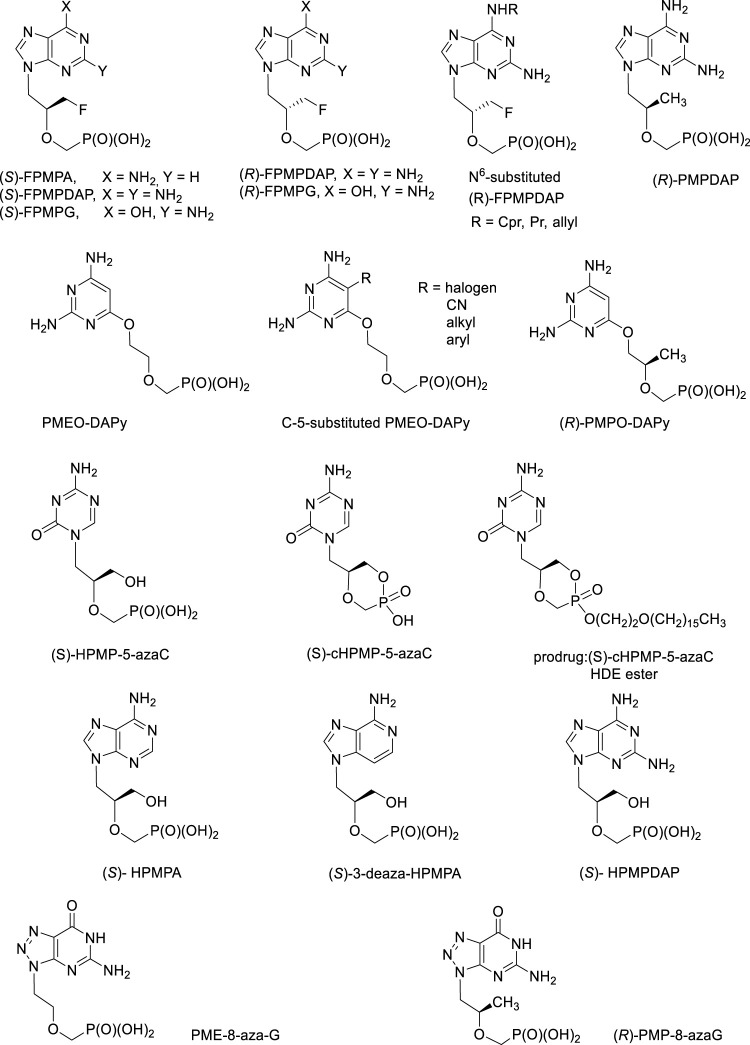
Biologically active acyclic nucleoside phosphonates still waiting for their opportunity for further development.

During more than 30 years of ANP research at the IOCB, hundreds of structures with excellent biological activities have been synthesized. An integral part of this story was always a personality of Erik De Clercq, Antonin Holy´s friend and collaborator leading all antiviral investigations and also author of many ANP reviews (De Clercq, 2009; De Clercq, 2011; De Clercq, 2013; De Clercq, 2019). Promising structures deserving further investigation are discussed below (Figure 11).

a) FPMP derivatives, i.e., (3-Fluoro-2-phosphonomethoxy)propyl derivatives.

This group of purine ANPs inhibits selectively retroviruses (HIV-1, HIV-2) and also HBV, with no effect towards other DNA viruses. The adenine derivative, (*S*)-FPMPA has better parameters *in vivo* compared to PMEA (adefovir) (Balzarini et al., 1991; Jindřich et al., 1993). Anti-HIV activity was also found in both enantiomers of FPMPDAP and FPMPG and some N^6^-substituted derivatives of (*R*)-FPMPDAP (cyclopropyl, propyl, allyl) (Baszczyňski and Janeba, 2013).

In the pyrimidine series, no antiviral activity was found but both enantiomers of the thymine derivative 1-[3-fluoro-2-phosphonomethoxy)propyl]thymine (FPMPT) have inhibitory activity towards thymidine phosphorylase, the enzyme playing the key role in the angiogenesis in tumors (Folkman and Shing, 1992; Esteban-Gamboa et al., 2000; Pomeisl et al., 2005). A study carried out on human purine nucleoside phosphorylase (PNP) revealed that the monophosphates derived from both enantiomers of FPMPG are its potent inhibitors. PNP is a purine salvage pathway enzyme catalysing the phosphorolysis of guanosine, inosine and 2′-deoxyguanosine to the corresponding purine base and ribose-1-phosphate or 2′-deoxyribose-1-phosphate, respectively. Pharmacological aspect of PNP inhibition is connected with the treatment of human T-cell proliferative disorders (Votruba et al., 2010)^101^.

b) (*R*)-PMP-derivatives, i.e., (*R*)-2-(Phosphonomethoxy)propyl derivatives.

The most active antiretroviral compound of this series, 2,6-diaminopurine analogue of tenofovir, (*R*)-PMPDAP, has not been thus far sufficiently studied, despite the fact that it is 10-fold more potent against HIV-1 compared to tenofovir *in vitro* and *in vivo* (Balzarini et al., 1993; Balzarini et al., 1996). It is also strongly active against animal retroviruses, especially feline immunodeficiency virus. Although strong conclusions cannot still be done, some studies revealed slightly positive (Vahlenkamp et al., 1995; Taffin et al., 2015). It is clear that the activity of (*R*)-PMPDAP *in vivo* warrants further investigations including synthesis of prodrugs (Krečmerová et al., 2013).

c) **“Open-ring” analogues** are ANPs having PME-, PMP or HPMP-grouping attached to the position 6 of the 2,4-diaminopyrimidine ring *via* oxygen atom. They mimic 2,6-diaminopurine ANPs with an open imidazole ring. Their antiviral activity is essentially identical to that of their parent compounds, including the enantiomeric specificity. It means that (*R*)-HPMPO-DAPy is a typical anti-DNA virus agent (similarly as cidofovir and other HPMP derivatives) whereas PMEO-DAPy, (*R*)-PMPO-DAPy a 5-substituted PMEO-DAPy inhibit retroviruses and HBV (Balzarini et al., 2002; Holý et al., 2002; Hocková et al., 2003; De Clercq et al., 2005; Herman et al., 2010). The most effective antiretroviral compounds are PMEO-5-methyl-DAPy and other 5-substituted PMEO-DAPy derivatives (Hocková et al., 2003). Compound PMEO-DAPy is active not only against retroviruses but also against many DNA viruses, especially herpesviruses affecting immunocompromised patients including those with HIV/AIDS. For this purpose we studied PMEO-DAPy also in the form of various structural types of prodrugs (Krečmerová et al., 2017).

d) HPMP-5-azaC (5-aza analogue of cidofovir), its cyclic form and prodrugs

1-(*S*)-[3-hydroxy-2-(phosphonomethoxy)propyl]-5-azacytosine (HPMP-5-azaC) has been developed as less toxic and more effective alternative to cidofovir. It shows potent and selective activity against all DNA viruses. The activity is comparable to cidofovir concerning herpes viruses (HSV-1, HSV-2) and vaccinia virus, and 2 to 7 times more active against varicella zoster virus (VZV), human cytomegalovirus (HCMV), human herpesvirus type 6 (HHV-6) and adenovirus (Ad2), with generally lower cytotoxicity (Krečmerová et al., 2007a). The prodrug form, hexadecyloxyethyl ester of cyclic HPMP-5-azaC revealed extremely high values of anti-DNA virus activities including imposing selectivity indices on the order of thousands, e.g., 1,160 for herpes simplex virus (HSV-1), ≥5,800 for varicella zoster virus (VZV) or ≥24,600 for human cytomegalovirus (HCMV) (Krečmerová et al., 2007b). Unfortunately, HPMP-5-azaC has rather complicated metabolic profile due to instability of the 5-azacytosine ring and the compound was finally not advanced to clinical investigations (Dračínský et al., 2008; Naesens et al., 2008).

e) HPMPA, HPMPDAP and aza/deaza purine base analogues

(*S*)-Enantiomers of HPMPA, HPMPDAP and 3-deazaHPMPA are compounds strongly active against DNA viruses, especially herpesviruses but their therapeutic possibilities are so far underexplored. Some of them, e.g., HPMPDAP could be useful for veterinary medicine for their effects towards African swine fever virus (Gil-Fernandez et al., 1987; Holý, 2003). First experiments with HPMPDAP performed in mice were successful. Unfortunately, application of free HPMPDAP to pigs caused serious side effects—the compound was strongly toxic. More research concerning dosing and formulation must be done but it is clear that future investigations need to be performed with prodrug forms (Roels et al., 2013).

**FIGURE 12:**
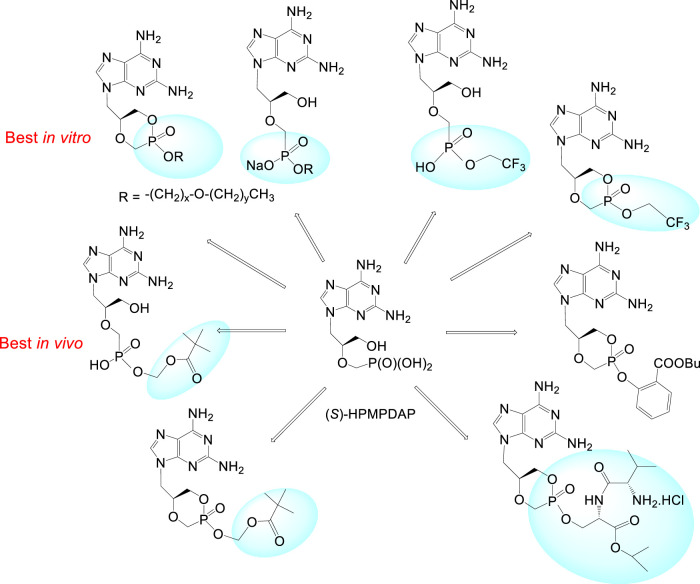
Prodrugs derived from (*S*)-HPMPDAP as antipox virus agents.

Special effort has been also paid to investigation of (*S*)-HPMPDAP and its prodrugs as anti-pox virus agents. The collaborative project of our institute with Rega Institute and Gilead Sciences had to find a potential drug candidate against poxviruses for the purpose of bioterrorist attack with variola virus (Krečmerová et al., 2010). Large series of cyclic and acyclic HPMPDAP esters were prepared: alkoxyalkyl, POM, 2,2,2-(trifluoro)ethyl, butylsalicylyl, and prodrugs based on peptidomimetics. The most potent prodrugs *in vitro* (tested on vaccinia virus) were the alkoxyalkyl ester derivatives with 50% effective concentrations 400- to 600-fold lower than those of the parent compound. Nevertheless, further *in vivo* experiments selected finally as the best candidate the acyclic POM ester (Figure 12).

Compounds deserving further investigation are also ANPs active against herpesviruses so far lacking any treatment (e.g., human herpesvirus type 6, HHV-6). HHV-6 takes part in pathogenesis of exanthema subitum (roseola), lymphoproliferative diseases, chronic fatigue syndrome and it is known also a co-factor of AIDS. It causes severe complications (e.g., encephalitis) in immunocompromised patients. Testing of ANPs for activity against HHV-6 selected 3-deaza-HPMPA and its cyclic form as compounds with the highest activity and selectivity (Reymen et al., 1995). In the series of 8-azapurine analogues, PME-8-azaguanine and (*R*)-PMP-8-azaguanine are compounds with interesting antiretroviral potency (Holý et al., 1996).

#### 2.2.2 Development of New ANP Structures

New ANP structures modified in the aliphatic side chain and/or in the base moiety are currently being developed in many laboratories. The successful example is besifovir, 9-[2-cyclopropyl-2-(phosphonomethoxy)ethyl]-2-aminopurine. Its prodrug form, besifovir disoproxil is investigated for the HBV treatment in the Phase III of clinical trials (Jung et al., 2020; Kim et al., 2020).

Replacement of a natural nucleobase with another heterocycle has been already mentioned in antibacterial PME-2-aminothiazoles (chapter 1.1.5). Another example is a series of HPMP and PME derivatives bearing a 1,2,4-thiadiazole base moiety which was intended as a cytosine mimic. In contrast to HPMPC, none of these compounds has antiviral activity but they were found potent inhibitors of cysteine dependent enzymes, such as Cathepsine K and glycogen synthase kinase 3β (Pomeislová et al., 2021).

Many side-chain modified ANPs are investigated as potent antiparasitic agents (see chapter 1.2.4.)

#### 2.2.3 New Prodrugs

Prodrugs are pharmacologically inactive compounds which are transformed *in vivo* to active drugs *via* metabolic and/or chemical processes occurring in the body. They are developed to optimize pharmacological properties of parent drugs. It is estimated that around 10% of all marketed drugs are prodrugs and their share in the new drug development is continuously increasing (Najjar and Karaman, 2019).

Prodrugs developed for acyclic nucleoside phosphonates can be categorized to the following groups (Pradere et al., 2014; Heidel and Dowd, 2019):

- Symmetrical phosphonate diesters—Alkyl, aryl, acyloxyalkyl (pivaloyloxymethyl, POM), alkoxycarbonyloxyalkyl (isopropoxycarbonyloxymethyl, POC), S-acylthioalkyl (SATE) (Benzaria et al., 1991).
- Asymmetric phosphonate diesters: HepDirect prodrugs (Erion et al., 2004; Reddy et al., 2008) Cyclosaligenyl (cycloSal) phosphonates (cyclic esters with variously substituted salicyl alcohol) (Meier, 2006; Meier and Balzarini, 2006). Despite an enormous amount of work in the area of cycloSal nucleosides, in the field of ANPs, only cycloSal-PMEA and cycloSal-(*R*)-PMPA were investigated (Meier et al., 2005).
- Phosphonate monoesters—internal cyclic monoesters (esters of cyclic HPMP derivatives, e.g., Figure 2, Figure 12) and monoesters -P(O)(OH)(OR) where R is alkyl, aryl, alkoxyalkyl (e.g., brincidofovir), POM, POC, alkyloxycarbonyl.
- Symmetric bisamidates—bisamidates with amino acid esters (e.g., rabacfosadine) (De Clercq, 2018).
- Asymmetric mixed ester/amidate/prodrugs—proTides (Cahard et al., 2004; Pradere et al., 2014; Heidel and Dowd, 2019).

Besides improving antiviral activity, cellular uptake and toxicity profile, some types of ANP prodrugs are also reported with respect to influence the antiviral activity spectrum of parent compounds. In the HPMP series, it concerns octadecyloxyethyl (ODE) monoesters derived of HPMPA. (*S*)-HPMPA is active exclusively against most double-stranded DNA viruses but has no *in vitro* effect against RNA viruses and retroviruses (Holý, 2003; De Clercq and Holý, 2005). Its opposite enantiomer, (*R*)-HPMPA is completely inactive against all viruses. Despite these facts, HDP and ODE monoesters of (*S*)-HPMPA were reported as active compounds against HCV (positive sense single-stranded RNA virus). Interestingly, even a prodrug derived from *R*-enantiomer was active against HCV (Wyles et al., 2009). Moreover, alkoxyalkyl esters of (*S*)-HPMPA and its 3-O-methyl derivative, (*S*)-MPMPA are potent inhibitors of HIV-1 replication (EC_50_ value of 7 nM for HDP ester of (*S*)-HPMPA) (Hostetler et al., 2006; Valiaeva et al., 2006). As expected, remarkable increase in antiviral activity against DNA viruses (HSV-1, HCMV, vaccinia, cowpox and ectromelia virus) was observed in ODE esters of (*S*)-HPMPA, (*S*)-HPMPG, (*S*)-HPMPDAP and its 6-cyclopropylamino analogue (*S*)-HPMP-cPrDAP. The two most active compounds against HSV-1 were ODE–(*S*)-HPMPA and ODE–(*S*)-HPMPC with subnanomolar EC_50_ values in cell cultures (Valiaeva et al., 2009).

Another example of influencing antiviral activity spectrum *via* synthesis of prodrugs are both enantiomers of 3-fluoro-2-(phosphonomethoxy)propyl]adenine (FPMPA) and other FPMP derivatives. These ANPs are effective antihuman immunodeficiency virus (HIV) agents, but have no activity against a wide range of DNA viruses. The introduction of a diamyl aspartate amidate motif together with a phenyl ester moiety at the phosphorus (*de facto* a special kind of proTides) not only enhanced antiviral potency against HIV (by a factor up to 1,500), but also against HBV. Interestingly, some of the synthesized compounds exhibited activity against DNA viruses, namely herpes viruses (Luo et al., 2017).

Despite of the huge number of prepared structures and promising *in vitro* or *in vivo* studies, only a few of them have been proceeded into clinical trials or clinics (POM, POC, HDP, proTides). It can be attributed to the difficulty of achievement of the right prodrug properties balance, particularly suitable chemical stability at physiological pH, rate of conversion, safety with respect to formed by-products, avoidance of stereoisomers, physical properties (ability to crystallize, solubility), and ability to synthesize substantial quantities. Therefore, search for new structural types of prodrug promoiety is highly desirable.

One such promising strategy is the concept of amino acid prodrugs (Krečmerová, 2017). Single amino acid ester prodrugs were reported in Herdewijn´s group for (*S*)-HPMPA and its cyclic form (Luo et al., 2018).

**FIGURE 13:**
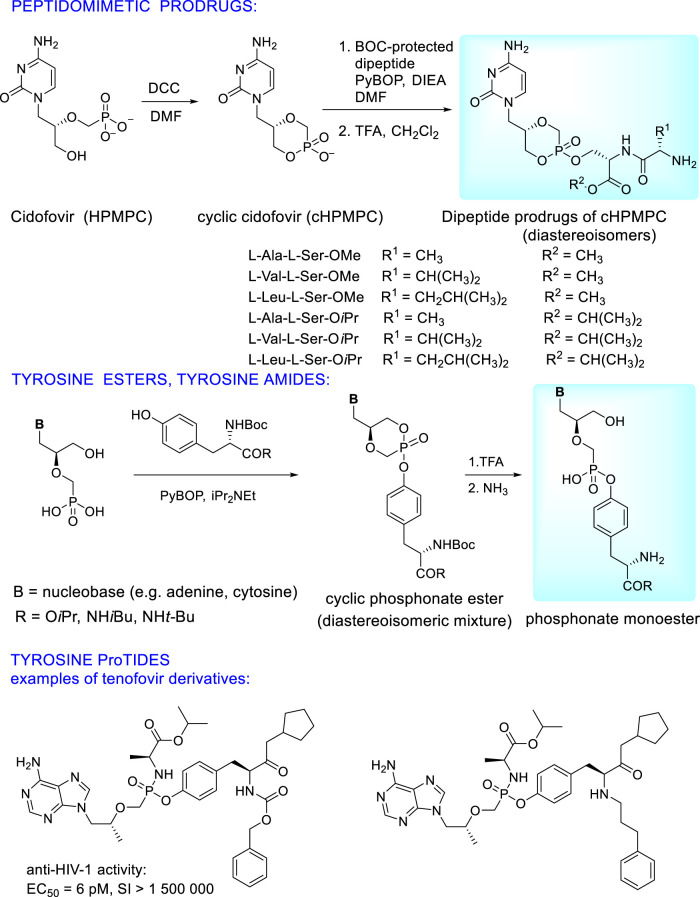
Examples of peptidomimetic and tyrosine based prodrugs of acyclic nucleoside phosphonates.

Extensive research is paid to prodrugs where the phosphonic acid residue is esterified by a hydroxyl group of hydroxyl amino acids (serine, threonine, tyrosine) which can be used either as single amino acid or as a component of small peptides (dipeptides and tripeptides). To circumvent the problem of the low permeation of peptides through the cell membranes and enzymatic instability in the gastrointestinal tract, structural modifications to form peptidomimetics are the solution. These modifications consist in esterification of a carboxyl group or its transformation. This approach enables wide spectrum of “fine tuning” of pharmacokinetic properties. Additionally, introduction of D-configurated N-terminal amino acids to the dipeptide increases enzymatic stability of the prodrug and its uptake to plasma (Eriksson et al., 2008; Peterson et al., 2011). Extensive research finally showed the best pharmacokinetic profiles in single amino acid prodrugs, especially tyrosine derivatives (Krylov et al., 2013; Zakharova et al., 2011; Williams et al., 2011). Synthesis and structures of peptidomimetic and tyrosine based ANP prodrugs are depicted in Figure 13 on the example of cidofovir. Analogous prodrugs were prepared also from (*S*)-HPMPA (Zakharova et al., 2011). They have excellent antiviral activity against DNA viruses (HCMV, poxviruses, HSV-1) without any cytotoxicity. Activity data of the cyclic forms are not dependent on stereochemistry at the phosphorus atom (EC_50_ for R_P ≈_S_P)_. Oral bioavailability in mice was 8-10x higher than that of parent phosphonates (39% vs. <5%) while the tyrosine alkylamide esters had better stability than carboxylate ester derivatives (Zakharova et al., 2011; Krylov et al., 2013).

Significant results have been recently published using tyrosine in synthesis of ANP proTides whose phenyl group is replaced with a modified (*S*)-tyrosine moiety (Figure 13). This approach applied to tenofovir leads to compounds with substantially increased potency and selectivity index towards HIV as well as HBV compared to tenofovir alafenamide. It is caused by their high cellular uptake and rapid cleavage to the parent drug tenofovir in the target cells (Kalčic et al., 2021).

#### 2.2.4 New Targets, New Applications: Development of ANPs as Antiparasitic Agents

Various purine ANPs have remarkable antiprotozoal activity. The therapeutic potential for the treatment of African trypanosomiasis was identified originally in (*S*)-HPMPA and (*S*)-HPMPDAP. The compounds revealed inhibitory effects against *Trypanosoma brucei brucei* both *in vitro* and *in vivo* while inhibitory activity against *Trypanosoma congolense* was found in PMEDAP (Kaminsky et al., 1994; Kaminsky et al., 1996). Recent studies selected as the best inhibitors 6-oxopurine ANPs further modified in the side chain (branched nitrogen containing derivatives, bisphosphonates and C-1′-branched ANPs) (Doleželová et al., 2018; Teran, 2020; Doležalová et al., 2021; Kalčic et al., 2021).

Extensive research is currently paid to ANPs as antimalarial agents. The parasitic 6-oxopurine phosphoribosyltransferases (PRTase) have been shown to be drug targets to inhibit malarial parasites (*Plasmodium falciparum*, *Plasmodium vivax*). 6-Oxopurine PRTases are present in all organisms. For a drug candidate, high selectivity towards parasitic enzyme PfHGPRT (and low or no inhibition of the human HGPRT) is always required (De Jersey et al., 2011; Keough, et al., 2013a).

**FIGURE 14:**
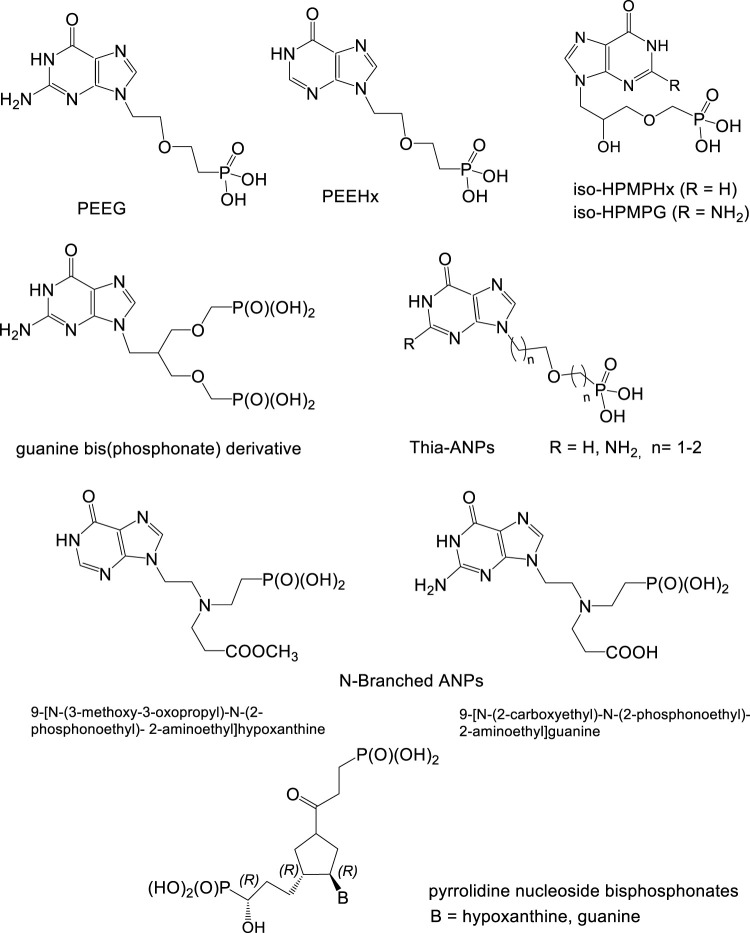
Acyclic nucleoside phosphonates as potential antimalarial agents.

Effective inhibitors of *Plasmodium* HGXPRT are guanine and hypoxanthine ANPs with the following arrangements of the side chain:

a) 2-(Phosphonoethoxy)ethyl derivatives (PEE derivatives: PEEG, PEEHx) (De Jersey et al., 2011; Keough, et al., 2013a).
b) 2-Hydroxy-3-phosphonomethoxypropyl (“iso-HPMP” derivatives) (Krečmerová et al., 2012).
c) Bisphosphonate structures formed by attachment of a second phosphonate group to the ANP scaffold. The most potent was found guanine derivative derived from iso-HPMPG by replacing its hydroxyl with a phosphonomethoxymethyl residue (Figure 14) (Keough, et al., 2013b).
d) N-Branched ANPs (aza-ANPs) containing a trisubstituted nitrogen in the side chain. The most potent inhibitors are 9-[N-(3-methoxy-3-oxopropyl)-N-(2-phosphonoethyl)-2-aminoethyl]hypoxanthine and 9-[N-(2-carboxyethyl)-N-(2-phosphonoethyl)-2-aminoethyl]guanine (Figure 14). The hypoxanthine derivative exhibits the highest ever reported selectivity for *Pf*HGXPRT compared to human HGPRT (Hocková et al., 2012).
e) Thia-ANPs—sulfur bridged acyclic nucleoside analogues, active against Plasmodium enzyme *Pf*HGXPRT in micromolar concentrations and highly selective compared to the human enzyme. Promising inhibitory data were found also in their phosphoramidate prodrugs (Klejch et al., 2019)

Besides above described guanine and hypoxanthine ANP inhibitors, there are also highly effective and selective “ANP-like“inhibitors with unnatural pyrrolidine base moiety (Keough et al., 2018). Purine metabolism was also identified as a ubiquitous factor in the physiology of various other pathogens including *Mycobacterium tuberculosis*, *Mycobacterium smegmatis* and other mycobacteria. The purine salvage enzyme HGPRT was found essential for *Mycobacterium tuberculosis* growth *in vitro*; however, its precise role in *M. tuberculosis* physiology is so far unclear. Membrane permeable prodrugs of HGPRT inhibitors arrest the growth of *M. tuberculosis* and represent potential new antituberculosis compounds (Knejzlík et al., 2020).

## 3 2-(Phosphonomethyl) Pentanedioic Acid, 2-PMPA

2-PMPA belongs to the group of phosphorus-based inhibitors of the enzyme glutamate carboxypeptidase II (GCPII) first identified by Jackson and Slusher in 1991 (Jackson at al., 1996; Jackson and Slusher, 2001). When first purified from the brain, Slusher et al. (Slusher et al., 1990) initially named the enzyme NAALADase for its substrate specificity for N-acetylated-alpha-linked acidic dipeptides; the enzyme has also been called N-acetylaspartylglutamate (NAAG) peptidase, prostate specific membrane antigen (PSMA) and folate hydrolase I (FOLH 1). The enzyme is a zinc metallopeptidase that hydrolyzes terminal glutamate from various substrates. The enzyme is a transmembrane glycoprotein which consists of six domains including the N-terminal cytoplasmic tail, a helical transmembrane region, and four large extracellular domains. The active site of GCPII where 2-PMPA and other competitive inhibitors bind is extracellularly facing (Bařinka et al., 2004).

In the nervous system GCPII converts N-acetylaspartylglutamate (NAAG), one of the most abundant peptides in the brain, to glutamate and N-acetylaspartate while in the jejunum its role also consists in the cleavage of pteroyolpoly-gamma-glutamate to folate and glutamate. In the prostate, GCPII is intensively studied as a both a prostate cancer biomarker and a target for radiotherapy (Jones et al., 2020; Zhang et al., 2021). More recently the Slusher team identified that GCPII enzymatic activity is highly upregulated in patient biopsies with inflammatory bowel disease (IBD) and inhibition of this upregulated activity provides therapeutic benefit in preclinical IBD models (Rais et al., 2016; Date et al., 2017; Peters et al., 2019; Peters et al., 2022).

Overexpression of GCPII in the brain leads to reduced NAAG and excess extracellular glutamate which can be pathogenic. Thus, inhibitors of GCPII have been investigated as therapeutic agents for disorders arising from excess glutamatergic transmission (Vornov et al., 2016; Neale and Yamamoto, 2020). Specifically, GCPII inhibitors have been developed as potential therapeutics for the treatment of neuropathic pain (Vornov et al., 2013), peripheral neuropathy (Zhang et al., 2002; Zhang et al., 2006), stroke (Slusher et al., 1999), amyotrophic lateral sclerosis (Ghadge et al., 2003; Tallon et al., 2022), multiple sclerosis (Rahn et al., 2012; Hollinger et al., 2022), schizophrenia (Olszewski et al., 2012), epilepsy (Luszczki et al., 2006), traumatic brain injury (Feng et al., 2011; Gurkoff et al., 2013), addiction (McKinzie et al., 2000; Xi et al., 2010), cognition (Janczura et al., 2013), and perinatal injury (Zhang et al., 2016).

**FIGURE 15:**
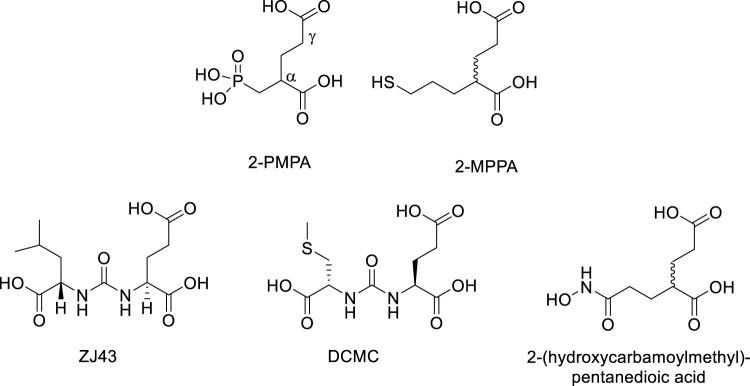
Representative examples of GCPII inhibitors.

GCPII inhibitors developed so far are polar compounds with structural similarity to NAAG and glutamate. In general, they contain a dicarboxylic acid moiety which binds to the C-terminal glutamate recognition site of GCPII and a zinc-binding group which engages one or both zinc atoms at the active site (Ferraris et al., 2012). The most potent classes belong to phosphonates such as 2-PMPA (Jackson at al., 1996), thiols such as 2-(3-mercaptopropyl)pentane-dioic acid (2-MPPA) (Majer et al., 2003), ureas such as N-[N-[(S)]-1,3-dicarboxypropyl]carbamoyl]-L-leucine (ZJ43) (Olszewski et al., 2017) and (N-[N-[(S)-1,3-dicarboxypropyl]carbamoyl]methyl-L-cysteine (DCMC) (Foss et al., 2005), and hydroxamates such as 2-(hydroxycarbamoylmethyl)pentanedioic acid (Stoermer et al., 2003) (Figure 15). Unfortunately, these compounds, with the exception of thiols, have low membrane permeability and oral bioavailability. The thiol inhibitor 2-MPPA advanced to clinical studies (van der Post et al., 2005). It was safe and well tolerated in two Phase 1 studies but subsequent immunological toxicities were observed in chronic GLP primate studies, halting its development. The toxicity was attributed to the thiol nature of the compound, not its GCPII inhibiting activity as it is well documented that thiol-containing drugs can elicit immune hypersensitivity reactions (Jaffe, 1986; Smith et al., 1989; Friedmann et al., 2003).

2-PMPA is one of the most potent GCPII inhibitors identified to date and has been found to have broad and robust neuroprotective and analgesic effects in many preclinical studies conducted by multiple independent laboratories (for review see: Barinka et al., 2012; Vornov et al., 2016; Nonaka et al., 2017). In addition to CNS diseases, GCPII provides metabolic substrates for cancer growth so its inhibition may provide anticancer activity (Kaittanis et al., 2018; Nguyen et al., 2019). In fact, a recent study showed the therapeutic potential of 2-PMPA in the treatment of glioblastoma (Gao et al., 2021). New opportunities may also arise using 2-PMPA as a nephron-protective strategy in PSMA-targeted prostate cancer radiotherapy (Kratochwil et al., 2015; Chatalic et al., 2016) or use of 2-PMPA as an inhibitor of cytosolic carboxypeptidases (Wang et al., 2020). Despite its proven utility in multiple preclinical models, clinical use of 2-PMPA remains problematic due to its poor pharmacokinetic profile including low cellular uptake, low oral bioavailability, and minimal brain penetration caused by its strongly polar character. Given this, the IOCB and Johns Hopkins Drug Discovery collaborative team decided to focus on synthesizing 2-PMPA prodrugs (Majer et al., 2016; Dash et al., 2019).

2-PMPA prodrugs were synthesised using two different strategies. The first strategy was focused on enhancing its oral bioavailability by covering its charge functionalities using FDA approved promoeities such as pivaloyloxymethyl (POM), alkoxycarbonyloxyalkyl (POC), and 5-methyl-2-oxo-1,3-dioxol-4-yl)methyl (ODOL) known to be activated *via* esterase enzymes expressed in intestines, plasma and liver (van Gelder et al., 2002; Rautio et al., 2008). The second strategy was focused on designing prodrugs for enhanced CNS penetration employing intranasal delivery methods (Rais et al., 2015; Nedelcovych et al., 2017).

**FIGURE 16:**
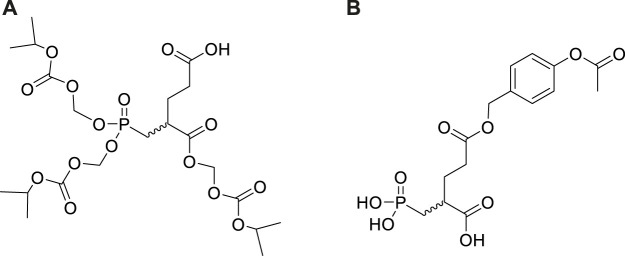
Structures of tris-POC 2-PMPA **(A)** and γ-(4-acetoxybenzyl)-2-PMPA **(B)**.

To develop orally available 2-PMPA prodrugs, our initial strategy was to cover only the phosphonate with hydrophobic moieties (POM, POC) keeping the *α*—and *γ* -carboxylates unsubstituted. Unfortunately, these derivatives were chemically unstable and exhibited low permeability. Addition of various *α*,*γ*-carboxylic diesters and α-monoesters to the bis-POC/POM derivatives enhanced their chemical stability but these mixed esters were too stable *in vivo*, resulting in minimal release of 2-PMPA. Iterative medicinal chemistry and pharmacokinetic efforts led to identification of tris-POC-2-PMPA (Figure 16) designed by introducing POC groups on both the phosphonate and the α-carboxylate. (Majer et al., 2016). In mice, oral tris-POC-2-PMPA provided sustained levels of 2-PMPA for over 4 h, with >20 fold enhancement in total 2-PMPA exposure when compared to orally administered 2-PMPA at a molar equivalent dose. The substantial oral exposure was subsequently confirmed in a beagle dog. The results provided the first example of orally bioavailable prodrugs of phosphonate based GCPII inhibitors and provided a roadmap for the design and development of other prodrugs from this potent class of compounds.

**FIGURE 17:**
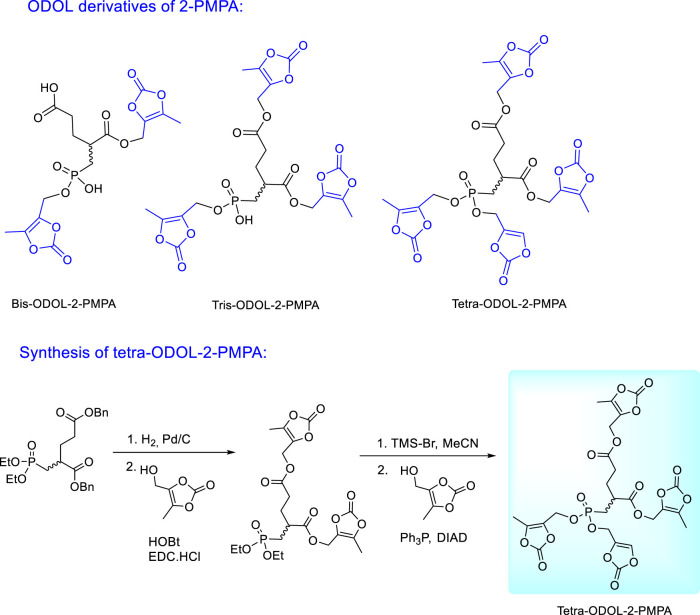
Synthesis and structures of (5-methyl-2-oxo-1,3-dioxol-4-yl)methyl (ODOL) ester prodrugs of 2-PMPA.

Encouraging results with tris-POC-2-PMPA stimulated further efforts in investigation and optimization of 2-PMPA prodrugs which resulted in the synthesis of (5-methyl-2-oxo-1,3- dioxol-4-yl)methyl (ODOL) derivatives (Figure 17). ODOL promoieties have been applied to enhance oral absorption of several FDA-approved drugs including olmesartan and azilsartan medoxomil (Brousil and Burke, 2003; Babu et al., 2009; Garaga et al., 2015). 2-PMPA derivatives masked with two, three, or four ODOL groups were synthesised and evaluated for *in vitro* stability and *in vivo* pharmacokinetics in mice and dogs (Dash et al., 2019). All prodrugs were found to be moderately stable at physiological pH, but rapidly hydrolysed in plasma and liver microsomes by the action of ubiquitous esterase enzymes. Like tris-POC-2-PMPA, ODOL prodrugs increased 2-PMPA plasma and brain exposures. The tetra-ODOL-2PMPA prodrug was the best and demonstrated a remarkable 80-fold enhancement in exposure versus oral 2-PMPA. In dogs, relative to orally administered 2-PMPA, the compound delivered a 44-fold enhanced 2-PMPA plasma exposure.

Unfortunately, while high plasma exposures were achieved with orally administered tris-POC-2-PMPA and tetra-ODOL-2-PMPA, the brain/plasma ratio remained low (<5%) due to their rapid conversion by plasma and liver enzymes. To further enhance brain exposures and brain/plasma ratio for use as a CNS therapeutic, we designed additional 2-PMPA prodrugs for intranasal (IN) administration by masking its γ-carboxylate. When compared to IN delivered 2-PMPA at 1 h post dose, *γ*-(4-acetoxybenzyl)-2-PMPA [Compound 1 in: Nedelcovych et al., 2017 and Figure 16)] resulted in enhanced delivery of 2-PMPA delivery to both plasma (4.1-fold) and brain (11-fold). The combined prodrug and IN delivery strategies are currently being employed with other inhibitors in an attempt to identify a clinically viable candidate.

The synthesis of tetra-ODOL-2-PMPA from 2-PMPA dibenzyl diethyl ester utilizes different reactivity of carboxylic and phosphonate esters towards bromotrimethylsilane. Carboxylic benzyl esters are deprotected first by catalytic hydrogenation and replaced by the ODOL moieties. The following deprotection of phosphonate ethyl esters with bromotrimethylsilane proceeds with the preservation of ODOL esters in the carboxylic groups which enables subsequent esterification of the phosphonate groups with 4-(hydroxymethyl)-5-methyl-1,3-dioxol-2-one (Figure 17).

## Conclusion

Phosphonates have unique and irreplaceable position in drug design and development due to their increased metabolic stability and bioisostericity with phosphates. The ability to interact with various enzymes and influence diverse metabolic pathways in the body is a benefit usable practically in all areas of medicine. The main research challenges on their way from chemistry to clinics are formulation strategies and/or transformation to prodrugs due to their low bioavailability related to a strongly polar character.

## Glossary

ACT: adenylate cyclase toxin
ANP: acyclic nucleoside phosphonate
cPrPMEDAP: 9-[(2-phosphonomethoxy)ethyl]-N^6^-cyclopropyl-2,6-diaminopurine
DAPy: 2,4-diaminopyrimidine
DCMC: (N-[N-[(S)-1,3-dicarboxypropyl]carbamoyl]methyl-L-cysteine
DIAD: diisopropyl azodicarboxylate
EF: edema factor
GCPII: glutamate carboxypeptidase II
FPMP: 3-fluoro-2-(phosphonomethoxy)propyl]
HBV: hepatitis B virus
HCMV: human cytomegalovirus
HCV: hepatitis C virus
HDP: hexadecyloxypropyl
HHV-6: human herpesvirus type 6
HPMP: 3-hydroxy-2-(phosphonomethoxy)propyl]
HPMPA: 9-[3-hydroxy-2-(phosphonomethoxy)propyl]adenine
HPMPC: 1-[3-hydroxy-2-(phosphonomethoxy)propyl]cytosine
HPMPDAP: 9-[3-hydroxy-2-(phosphonomethoxy)propyl]-2,6-diaminopurine
HSV-1: herpes simplex virus type 1
IBD: inflammatory bowel disease
LPS: lipopolysaccharide
2-MPPA: 2-(3-mercaptopropyl)pentane-dioic acid
NAAG: N-acetylaspartylglutamate
ODE: octadecyloxyethyl
PEE: 2-(phosphonoethoxy)ethyl
PfHGPRT: *Plasmodium falciparum* hypoxanthine-guanine-xanthine phosphoribosyltransferase
PME: 2-(phosphonomethoxy)ethyl
PMEA: 9-[2-(phosphonomethoxy)ethyl]adenine
PMEDAP: 9-[2-(phosphonomethoxy)ethyl]-2,6-diaminopurine
PMEG: 9-[2-(phosphonomethoxy)ethyl]guanine
PMEO-DAPy: 6-[2-(phosphonomethoxy)ethoxy]-2,4-diaminopyrimidine
PMP: 2-(phosphonomethoxy)propyl
PMPA: 9-[2-(phosphonomethoxy)propyl]adenine
2-PMPA: 2-(phosphonomethyl) pentanedioic acid
POM: pivaloyloxymethyl
POC: isopropoxycarbonyloxymethyl
PRTase: phosphoribosyltransferase
PSMA: prostate specific membrane antigen
ODOL: (5-methyl-2-oxo-1,3-dioxol-4-yl)methyl
SATE: S-acyl-2-thioethyl
TDF: tenofovir disoprovil fumarate

## References

[B1] Alcamo A. M. Wolf M. S. Alessi L. J. Chong H. J. Green M. Williams J. V. (2020). Successful Use of Cidofovir in an Immunocompetent Child with Severe Adenoviral Sepsis. Pediatrics 145 (1), 1632. 10.1542/peds.2019-1632 PMC6939840 31826930

[B2] Andrei G. Snoeck R. (2010). Cidofovir Activity against Poxvirus Infections. Viruses-Basel 2 (12), 2803–2830. 10.3390/v2122803 PMC3185586 21994641

[B3] Andrei G. Topalis D. De Schutter T. Snoeck R. (2015). Insights into the Mechanism of Action of Cidofovir and Other Acyclic Nucleoside Phosphonates against Polyoma- and Papillomaviruses and Non-viral Induced Neoplasia. Antivir. Res. 114, 21–46. 10.1016/j.antiviral.2014.10.012 25446403

[B4] Arimilli M. N. Dougherty J. Cundy K. C. Bischofberger N. (1999). Orally Bioavailable Acyclic Nucleoside Phosphonate Prodrugs: Adefovir Dipivoxil and bis(POC)PMPA. Adv. Antivir. Drug Res. 3, 69–91. 10.1016/s1075-8593(99)80004-5

[B5] Arimilli M. N. Kim C. U. Dougherty J. Mulato A. Oliyai R. Shaw J. P. (1997). Synthesis, *In Vitro* Biological Evaluation and Oral Bioabailability of 9-[2-(phosphonomethoxy)propyl]adenine (PMPA) Prodrugs. Antivir. Chem. Chemother. 8 (6), 557–564. 10.1177/095632029700800610

[B6] Babu K. S. Reddy M. S. Tagore A. R. Reddy G. S. Sebastian S. Varma M. S. (2009). Efficient Synthesis of Olmesartan Medoxomil, an Antihypertensive Drug. Synth. Commun. 39 (2), 291–298. 10.1080/00397910802372558

[B7] Balzarini J. Aquaro S. Perno C.-F. Witvrouw M. Holý A. De Clercq E. (1996). Activity of the (R)-enantiomers of 9-(2-phosphonylmethoxypropyl) Adenine and 9-(2-Phosphonylmethoxypropyl)-2,6-Diaminopurine against Human Immunodeficiency Virus in Different Human Cell Systems. Biochem. Biophys. Res. Commun. 219 (2), 337–341. 10.1006/bbrc.1996.0234 8604988

[B8] Balzarini J. Holý A. Jindřich J. Dvořáková H. Hao Z. Snoeck R. (1991). 9-[(2RS)-3-fluoro-2-phosphonylmethoxypropyl] Derivatives of Purines: a Class of Highly Selective Antiretroviral Agents *In Vitro* and *In Vivo* . Proc. Natl. Acad. Sci. U. S. A. 88 (11), 4961–4965. 10.1073/pnas.88.11.4961 1711214 PMC51787

[B9] Balzarini J. Holý A. Jindrich J. Naesens L. Snoeck R. Schols D. (1993). Differential Antiherpesvirus and Antiretrovirus Effects of the (S) and (R) Enantiomers of Acyclic Nucleoside Phosphonates: Potent and Selective *In Vitro* and *In Vivo* Antiretrovirus Activities of (R)-9-(2-phosphonomethoxypropyl)-2,6-diaminopurine. Antimicrob. Agents Chemother. 37 (2), 332–338. 10.1128/AAC.37.2.332 8452366 PMC187663

[B10] Balzarini J. Pannecouque C. De Clercq E. Aquaro S. Perno C. F. Egberink H. (2002). Antiretrovirus Activity of a Novel Class of Acyclic Pyrimidine Nucleoside Phosphonates. Antimicrob. Agents Chemother. 46 (7), 2185–2193. 10.1128/AAC.46.7.2185-2193.2002 12069973 PMC127315

[B11] Bařinka C. Mlčochová P. Šácha P. Hilgert I. Majer P. Slusher B. S. (2004). Amino Acids at the N- and C-Termini of Human Glutamate Carboxypeptidase II Are Required for Enzymatic Activity and Proper Folding. Eur. J. Biochem. 271 (13), 2782–2790. 10.1111/j.1432-1033.2004.04209.x 15206943

[B12] Bařinka C. Rojas C. Slusher B. S. Pomper M. (2012). Glutamate Carboxypeptidase II in Diagnosis and Treatment of Neurologic Disorders and Prostate Cancer. Curr. Med. Chem. 19 (6), 856–870. 10.2174/092986712799034888 22214450 PMC3341092

[B13] Baszczyňski O. Janeba Z. (2013). Medicinal Chemistry of Fluorinated Cyclic and Acyclic Nucleoside Phosphonates. Med. Res. Rev. 33 (6), 1304–1344. 10.1002/med.21296 23893552

[B14] Beadle J. R. Hartline C. Aldern K. A. Rodriguez N. Harden E. Kern E. R. (2002). Alkoxyalkyl Esters of Cidofovir and Cyclic Cidofovir Exhibit Multiple-Log Enhancement of Antiviral Activity against Cytomegalovirus and Herpesvirus Replication *In Vitro* . Antimicrob. Agents Chemother. 46 (8), 2381–2386. 10.1128/AAC.46.8.2381-2386.2002 12121908 PMC127379

[B15] Benzaria S. Pelicano H. Johnson R. Maury G. Imbach J. L. Aubertin A. M. (1996). Synthesis, *In Vitro* Antiviral Evaluation, and Stability Studies of bis(S-Acyl-2-Thioethyl) Ester Derivatives of 9-[2-(phosphonomethoxy)ethyl]adenine (PMEA) as Potential PMEA Prodrugs with Improved Oral Bioavailability. J. Med. Chem. 39 (25), 4958–4965. 10.1021/jm960289o 8960556

[B16] Birkus G. Bam R. A. Willkom M. Frey C. R. Tsai L. Stray K. M. (2016). Intracellular Activation of Tenofovir Alafenamide and the Effect of Viral and Host Protease Inhibitors. Antimicrob. Agents Chemother. 60 (1), 316–322. 10.1128/AAC.01834-15 26503655 PMC4704186

[B17] Břehová P. Chaloupecká E. Česnek M. Skácel J. Dračínský M. Tloušťová E. (2021). Acyclic Nucleoside Phosphonates with 2-aminothiazole Base as Inhibitors of Bacterial and Mammalian Adenylate Cyclases. Eur. J. Med. Chem. 222, 113581. 10.1016/j.ejmech.2021.113581 34102377 PMC8373703

[B18] Břehová P. Šmídková M. Skácel J. Dračínský M. Mertlíková-Kaiserová H. Velasquez M. P. S. (2016). Design and Synthesis of Fluorescent Acyclic Nucleoside Phosphonates as Potent Inhibitors of Bacterial Adenylate Cyclases. ChemMedChem 11 (22), 2534–2546. 10.1002/cmdc.201600439 27775243 PMC5198786

[B19] Brousil J. A. Burke J. M. J. C. (2003). Olmesartan Medoxomil: an Angiotensin II-Receptor Blocker. Clin. Ther. 25 (4), 1041–1055. 10.1016/S0149-2918(03)80066-8 12809956

[B20] Cahard D. McGuigan C. Balzarini J. (2004). Aryloxy Phosphoramidate Triesters as Pro-tides. Mini Rev. Med. Chem. 4 (4), 371–378. 10.2174/1389557043403936 15134540

[B21] Česnek M. Jansa P. Šmídková M. Mertlíková-Kaiserová H. Dračínský M. Brust T. F. (2015). Bisamidate Prodrugs of 2-substituted 9- 2-(phosphonomethoxy)ethyl Adenine (PMEA, Adefovir) as Selective Inhibitors of Adenylate Cyclase Toxin from Bordetella Pertussis. ChemMedChem 10 (8), 1351–1364. 10.1002/cmdc.201500183 26136378

[B22] Česnek M. Šafránek M. Dračínský M. Tloušťová E. Mertlíková-Kaiserová H. Hayes M. P. (2022). Halogen-Dance-Based Synthesis of Phosphonomethoxyethyl (PME) Substituted 2-Aminothiazoles as Potent Inhibitors of Bacterial Adenylate Cyclases. ChemMedChem 17 (1), e202100568. 10.1002/cmdc.202100568 34636150 PMC8741643

[B23] Česnek M. Skácel J. Jansa P. Dračínský M. Šmídková M. Mertlíková-Kaiserová H. (2018). Nucleobase Modified Adefovir (PMEA) Analogues as Potent and Selective Inhibitors of Adenylate Cyclases from Bordetella Pertussis and Bacillus Anthracis. ChemMedChem 13 (17), 1779–1796. 10.1002/cmdc.201800332 29968968 PMC6415679

[B24] Chapman H. Kernan M. Prisbe E. Rohloff J. Sparacino M. Terhorst T. (2001). Practical Synthesis, Separation, and Stereochemical Assignment of the PMPA Pro-drug GS-7340. Nucleosides, Nucleotides, Nucleic Acids 20 (4-7), 621–628. 10.1081/NCN-100002338 11563079

[B25] Chatalic K. L. S. Heskamp S. Konijnenberg M. Molkenboer-Kuenen J. D. M. Franssen G. M. Clahsen-van Groningen M. C. (2016). Towards Personalized Treatment of Prostate Cancer: PSMA I&T, a Promising Prostate-specific Membrane Antigen-Targeted Theranostic Agent. Theranostics 6 (6), 849–861. 10.7150/thno.14744 27162555 PMC4860893

[B26] Chen W. Flavin M. T. Filler R. Xu Z. Q. (1996). An Improved Synthesis of 9-[2-(diethoxyphosphonomethoxy)ethyl]adenine and its Analogues with Other Purine Bases Utilizing the Mitsunobu Reaction. Nucleosides Nucleotides 15 (11-12), 1771–1778. 10.1080/07328319608002731

[B27] Cheng X. Cook G. P. Desai M. (2005). Phosphonates, Monophosphonamidates Bisphosphonamidates for the Treatment of Viral Diseases. U.S. Patent No WO2005066189 A1. Foster City, CA, United States: Gilead Sciences, Inc. Publication date 21. 07. 2005.

[B28] Cihlar T. Birkus G. Greenwalt D. E. Hitchcock M. J. M. (2002). Tenofovir Exhibits Low Cytotoxicity in Various Human Cell Types: Comparison with Other Nucleoside Reverse Transcriptase Inhibitors. Antivir. Res. 54 (1), 37–45. 10.1016/S0166-3542(01)00210-8 11888656

[B29] Clinical.trials.gov (2021). Brincidofovir. Available at: https://clinicaltrials.gov/ct2/results?term=brincidofovir&Search=Search .

[B30] Clinical.trials.gov (2014). GS-9219. Available at: http://clinicaltrials.gov/ct2/results?term=GS-9219&Search=Search .

[B31] Compton M. L. Toole J. J. Paborsky L. R. (1999). 9-(2-Phosphonylmethoxyethyl)-N-6- Cyclopropyl-2,6-Diaminopurine (Cpr-PMEDAP) as a Prodrug of 9-(2- Phosphonylmethoxyethyl)guanine (PMEG). Biochem. Pharmacol. 58 (4), 709–714. 10.1016/S0006-2952(99)00138-0 10413310

[B32] Cundy K. C. (1999). Clinical Pharmacokinetics of the Antiviral Nucleotide Analogues Cidofovir and Adefovir. Clin. Pharmacokinet. 36 (2), 127–143. 10.2165/00003088-199936020-00004 10092959

[B33] Dash R. P. Tichý T. Veeravalli V. Lam J. Alt J. Wu Y. (2019). Enhanced Oral Bioavailability of 2-(Phosphonomethyl)-Pentanedioic Acid (2-PMPA) from its (5-Methyl-2-Oxo-1,3-Dioxol-4-Yl)methyl (ODOL)-Based Prodrugs. Mol. Pharm. 16 (10), 4292–4301. 10.1021/acs.molpharmaceut.9b00637 31503493 PMC7978038

[B34] Date A. A. Rais R. Babu T. Ortiz J. Kanvinde P. Thomas A. G. (2017). Local Enema Treatment to Inhibit FOLH1/GCPII as a Novel Therapy for Inflammatory Bowel Disease. J. Control Release 263, 132–138. 10.1016/j.jconrel.2017.01.036 28159515 PMC5661937

[B35] De Clercq E. (2007). Acyclic Nucleoside Phosphonates: Past, Present and Future. Bridging Chemistry to HIV, HBV, HCV, HPV, Adeno-, Herpes-, and Poxvirus Infections: The Phosphonate Bridge. Biochem. Pharmacol. 73 (7), 911–922. 10.1016/j.bcp.2006.09.014 17045247

[B36] De Clercq E. Andrei G. Balzarini J. Leyssen P. Naesens L. Neyts J. (2005). Antiviral Potential of a New Generation of Acyclic Nucleoside Phosphonates, the 6-[2-(phosphonomethoxy)alkoxy]-2,4-Diaminopyrimidines. Nucleosides, Nucleotides Nucleic Acids 24 (5-7SI), 331–341. 10.1081/NCN-200059772 16247948

[B37] De Clercq E. (2009). Antiviral Drug Discovery: Ten More Compounds, and Ten More Stories (Part B). Med. Res. Rev. 29 (4), 571–610. 10.1002/med.20149 19219846

[B38] De Clercq E. (2019). Fifty Years in Search of Selective Antiviral Drugs. J. Med. Chem. 62 (16), 7322–7339. 10.1021/acs.jmedchem.9b00175 30939009

[B39] De Clercq E. Holý A. (2005). Acyclic Nucleoside Phosphonates: A Key Class of Antiviral Drugs. Nat. Rev. Drug Discov. 4 (11), 928–940. 10.1038/nrd1877 16264436

[B40] De Clercq E. (2018). Tanovea® for the Treatment of Lymphoma in Dogs. Biochem. Pharmacol. 154, 265–269. 10.1016/j.bcp.2018.05.010 29778492

[B41] De Clercq E. (2016). Tenofovir Alafenamide (TAF) as the Successor of Tenofovir Disoproxil Fumarate (TDF). Biochem. Pharmacol. 119, 1–7. 10.1016/j.bcp.2016.04.015 27133890

[B42] De Clercq E. (2013). The Acyclic Nucleoside Phosphonates (ANPs): Antonín Holy’s Legacy. Med. Res. Rev. 33 (6SI), 1278–1303. 10.1002/med.21283 23568857

[B43] De Clercq E. (2011). The Clinical Potential of the Acyclic (And Cyclic) Nucleoside Phosphonates. The Magic of the Phosphonate Bond. Biochem. Pharmacol. 82 (2), 99–109. 10.1016/j.bcp.2011.03.027 21501598

[B44] de Fraga R. S. Van Vaisberg V. Mendes L. C. A. Carrilho F. J. Ono S. K. (2020). Adverse Events of Nucleos(t)ide Analogues for Chronic Hepatitis B: a Systematic Review. J. Gastroenterol. 55 (5), 496–514. 10.1007/s00535-020-01680-0 32185517 PMC7188775

[B45] De Jersey J. Holý A. Hocková D. Naesens L. Keough D. T. Guddat L. W. (2011). 6-Oxopurine Phosphoribosyltransferase: A Target for the Development of Antimalarial Drugs. Curr. Top. Med. Chem. 11 (16), 2085–2102. 10.2174/156802611796575911 21619515

[B46] Deeks E. D. Perry C. M. (2010). Efavirenz/Emtricitabine/Tenofovir Disoproxil Fumarate Single-Tablet Regimen (Atripla (R)). A Review of its Use in the Management of HIV Infection. Drugs 70 (17), 2315–2338. 10.2165/11203800-000000000-00000 21080746

[B47] Doležalová E. Klejch T. Špaček P. Slapničková M. Guddat L. Hocková D. (2021). Acyclic Nucleoside Phosphonates with Adenine Nucleobase Inhibit Trypanosoma Brucei Adenine Phosphoribosyltransferase *In Vitro* . Sci. Rep. 11, 13317. 10.1038/s41598-021-91747-6 34172767 PMC8233378

[B48] Doleželová E. Terán D. Gahura O. Kotrbová Z. Procházková M. Keough D. (2018). Evaluation of the Trypanosoma Brucei 6-oxopurine Salvage Pathway as a Potential Target for Drug Discovery. PLoS Negl. Trop. Dis. 12 (2), e0006301. 10.1371/journal.pntd.0006301 29481567 PMC5843355

[B49] Dračínský M. Krečmerová M. Holý A. (2008). Study of Chemical Stability of Antivirally Active -5azacytosine Acyclic Nucleoside Phosphonates Using NMR Spectroscopy. Bioorg. Med. Chem. 16 (14), 6778–6782. 10.1016/j.bmc.2008.05.058 18554916

[B50] Drugs.com (2021). Cidofovir. Available at: https://www.drugs.com/monograph/cidofovir.html (Accessed June 9, 2021).

[B51] Dunning J. Kennedy S: B. Antierens A. Whitehead J. Ciglenecki I. Carson G. (2016). Experimental Treatment of Ebola Virus Disease with Brincidofovir. Plos One 11 (9), e0162199. 10.1371/journal.pone.0162199 27611077 PMC5017617

[B52] Elanco (2021). Tanovea®(rabacfosadine for injection). Available at: https://www.elanco.us/products-services/dogs/tanovea.

[B53] Eriksson U. Peterson L. W. Kashemirov B. A. Hilfinger J. M. Drach J. C. Borysko K. Z. (2008). Serine Peptide Phosphoester Prodrugs of Cyclic Cidofovir: Synthesis, Transport and Antiviral Activity. Mol. Pharm. 5 (4), 598–609. 10.1021/mp8000099 18481868 PMC2629803

[B54] Erion M. D. Reddy K. R. Boyer S. H. Matelich M. C. Gornez-Galeno J. Lemus R. H. (2004). Design, Synthesis, and Characterization of a Series of Cytochrome P-450 3A-Activated Prodrugs (HepDirect Prodrugs) Useful for Targeting Phosph(on)ate-Based Drugs to the Liver. J. Am. Chem. Soc. 126 (16), 5154–5163. 10.1021/ja031818y 15099098

[B55] Esteban-Gamboa A. Balzarini J. Esnouf R. De Clercq E. Camarasa M. J. Perez-Perez M. J. (2000). Design, Synthesis, and Enzymatic Evaluation of Multisubstrate Analogue Inhibitors of Escherichia coli Thymidine Phosphorylase. J. Med. Chem. 43 (5), 971–983. 10.1021/jm9911377 10715161

[B56] F. D. A. (2021). FDA . Available at: https://www.fda.gov/drugs/news-events-human-drugs/fda-approves-drug-treat-smallpox (Accessed June 4, 2021).

[B57] Feng J. F. Van K. C. Gurkoff G. G. Kopriva C. Olszewski R. T. Song M. (2011). Post-injury Administration of NAAG Peptidase Inhibitor Prodrug, PGI-02776, in Experimental TBI. Brain Res. 1395, 62–73. 10.1016/j.brainres.2011.04.022 21565332 PMC3105192

[B58] Ferraris D. V. Shukla K. Tsukamoto T. (2012). Structure-activity Relationships of Glutamate Carboxypeptidase II (GCPII) Inhibitors. Curr. Med. Chem. 19 (9), 1282–1294. 10.2174/092986712799462658 22304717

[B59] Folkman J. Shing Y. (1992). Angiogenesis. J. Biol. Chem. 267 (16), 10931–10934. 10.1016/s0021-9258(19)49853-0 1375931

[B60] Fontaine H. Vallet-Pichard A. Chaix M.-L. Currie G. Serpaggi J. Verkarre V. (2005). Efficacy and Safety of Adefovir Dipivoxil in Kidney Recipients, Hemodialysis Patients, and Patients with Renal Insufficiency. Transplantation 80 (8), 1086–1092. 10.1097/01.tp.0000178305.39231.a2 16278590

[B61] Foss C. A. Mease R. C. Fan H. Wang Y. Ravert H. T. Dannals R. F. (2005). Radiolabeled Small-Molecule Ligands for Prostate-specific Membrane Antigen: *In Vivo* Imaging in Experimental Models of Prostate Cancer. Clin. Cancer Res. 11 (11), 4022–4028. 10.1158/1078-0432.CCR-04-2690 15930336

[B62] Friedmann P. S. Lee M. S. Friedmann A. C. Barnetson R. S. C. (2003). Mechanisms in Cutaneous Drug Hypersensitivity Reactions. Clin. Exp. Allergy 33 (7), 861–872. 10.1046/j.1365-2222.2003.01718.x 12859440

[B63] Gao Y. Zheng H. Li L. Feng M. Chen X. Hao B. (2021). Prostate-Specific Membrane Antigen (PSMA) Promotes Angiogenesis of Glioblastoma through Interacting with ITGB4 and Regulating NF-Κb Signaling Pathway. Front. Cell Dev. Biol. 9, 598377. 10.3389/fcell.2021.598377 33748101 PMC7969793

[B64] Garaga S. Misra N. C. Raghava Reddy A. V. Prabahar K. J. Takshinamoorthy C. Sanasi P. D. (2015). Commercial Synthesis of Azilsartan Kamedoxomil: An Angiotensin II Receptor Blocker. Org. Process Res. Dev. 19 (4), 514–519. 10.1021/op500357r

[B65] Ghadge G. D. Slusher B. S. Bodner A. Canto M. D. Wozniak K. Thomas A. (2003). Glutamate Carboxypeptidase II Inhibition Protects Motor Neurons from Death in Familial Amyotrophic Lateral Sclerosis Models. Proc. Natl. Acad. Sci. U. S. A. 100 (16), 9554–9559. 10.1073/pnas.1530168100 12876198 PMC170956

[B66] Gi R. E. A. T. P. Dietz A. Djukic V. Eckel H. E. Friedrich G. Golusinski W. (2012). Treatment of Recurrent Respiratory Papillomatosis and Adverse Reactions Following Off-Label Use of Cidofovir (Vistide^®^). Eur. Arch. Otorhinolaryngol. 269 (2), 361–362. 10.1007/s00405-011-1804-7 22020697 PMC3259328

[B67] Gibb D. M. Kizito H. Russell E. C. Chidziva E. Zalwango E. Nalumenya R. (2012). Pregnancy and Infant Outcomes Among HIV-Infected Women Taking Long-Term ART with and without Tenofovir in the DART Trial. Plos Med. 9 (5), e1001217. 10.1371/journal.pmed.1001217 22615543 PMC3352861

[B68] Gil-Fernandez C. Garcia-Villalon D. De Clercq E. Rosenberg I. Holý A. (1987). Phosphonylmethoxyalkylpurines and –pyrimidines as Inhibitors of African Swine Fever Virus Replication *In Vitro* . Antivir. Res. 8 (5-8), 273–281. 10.1016/S0166-3542(87)80005-0 3451699

[B69] Gilead (2022). Medicine. Available at: https://www.gilead.com/science-and-medicine/medicines .

[B70] Göbel R. Richter F. Weichmann H. (1992). Synthesis and Reactivity of Methylene Bridged Diphosphoryl Compounds. Phosphorus, Sulfur Silicon 73 (1-4), 67–80. 10.1080/10426509208034433

[B71] Gurkoff G. G. Feng J. F. Van K. C. Izadi A. Ghiasvand R. Shahlaie K. (2013). NAAG Peptidase Inhibitor Improves Motor Function and Reduces Cognitive Dysfunction in a Model of TBI with Secondary Hypoxia. Brain Res. 1515, 98–107. 10.1016/j.brainres.2013.03.043 23562458 PMC3672358

[B72] Hatse S. Naesens L. De Clercq E. Balzarini J. (1999). N(6)-Cyclopropyl-PMEDAP: A Novel Derivative of 9-(2-Phosphonylmethoxyethyl)-2-6-Diaminopurine (PMEDAP) with Distinct Metabolic, Antiproliferative, and Differentiation-Inducing Properties. Biochem. Pharmacol. 58 (2), 311–323. 10.1016/S0006-2952(99)00091-X 10423173

[B73] Heidel K. M. Dowd C. S. (2019). Phosphonate Prodrugs: an Overview and Recent Advances. Future Med. Chem. 11 (13), 1625–1643. 10.4155/fmc-2018-0591 31469328 PMC6722485

[B74] Herman B. D. Votruba I. Holý A. Sluis-Cremer N. Balzarini J. (2010). The Acyclic 2,4-diaminopyrimidine Nucleoside Phosphonate Acts as a Purine Mimetic in HIV-1 Reverse Transcriptase DNA Polymerization. J. Biol. Chem. 285 (16), 12101–12108. 10.1074/jbc.M109.096529 20164190 PMC2852949

[B75] Hézode C. Chevaliez S. Bouvier-Alias M. Roudot-Thoraval F. Brillet R. Zafrani E. S. (2007). Efficacy and Safety of Adefovir Dipivoxil 20 Mg Daily in HBeAg-Positive Patients with Lamivudine-Resistant Hepatitis B Virus and a Suboptimal Virological Response to Adefovir Dipivoxil 10 Mg Daily. J. Hepatol. 46 (5), 791–796. 10.1016/j.jhep.2007.01.018 17321635

[B76] Hocková D. Holý A. Masojídková M. Andrei G. Snoeck R. De Clercq E. (2003). 5-Substituted-2,4-diamino-6-[2-(phosphonomethoxy)ethoxy]pyrimidines - Acyclic Nucleoside Phosphonate Analogues with Antiviral Activity. J. Med. Chem. 46 (23), 5064–5073. 10.1021/jm030932o 14584956

[B77] Hocková D. Keough D. T. Janeba Z. Wang T.-H. De Jersey J. Guddat L. W. (2012). Synthesis of Novel N-Branched Acyclic Nucleoside Phosphonates as Potent and Selective Inhibitors of Human Plasmodium Falciparum and Plasmodium Vivax 6-oxopurine Phosphoribosyltransferases. J. Med. Chem. 55 (13), 6209–6223. 10.1021/jm300662d 22725979

[B78] Hollinger K. R. Sharma A. Tallon C. Lovell L. Thomas A. G. Zhu X. (2022). Dendrimer-2PMPA Selectively Blocks Upregulated Microglial GCPII Activity and Improves Cognition in a Mouse Model of Multiple Sclerosis. Nanotheranostics 6 (2), 126–142. 10.7150/ntno.63158 34976589 PMC8671953

[B79] Holý A. Dvořáková H. Jindřich J. Masojídková M. Buděšínský M. Balzarini J. (1996). Acyclic Nucleotide Analogs Derived from 8-Azapurines: Synthesis and Antiviral Activity. J. Med. Chem. 39 (20), 4073–4088. 10.1021/jm960314q 8831773

[B80] Holý A. Dvořáková H. Masojídková M. (1995). Synthesis of Enantiomeric N-(2-phosphonomethoxypropyl) Derivatives of Heterocyclic Bases. 2. Synthon Approach. Collect. Czech Chem. Commun. 60 (8), 1390–1409. 10.1135/cccc19951390

[B81] Holý A. Günter J. Dvořáková H. Masojídková G. Andrei G. Snoeck R. (1999). Structure-antiviral Activity Relationship in the Series of Pyrimidine and Purine N-[2-(2- Phosphonomethoxy)ethyl] Nucleotide Analogues. 1. Derivatives Substituted at the Carbon Atoms of the Base. J. Med. Chem. 42 (12), 2064–2086. 10.1021/jm9811256 10377214

[B82] Holý A. (2003). Phosphonomethoxyalkyl Analogs of Nucleotides. Curr. Pharm. Des. 9 (31), 2567–2592. 10.2174/1381612033453668 14529543

[B83] Holý A. Rosenberg I. Dvořáková H. (1989). Synthesis of N-(2-phosphonylmethoxyethyl) Derivatives of Heterocyclic Bases. Collect. Czech Chem. Commun. 54 (8), 2190–2210. 10.1135/cccc19892190

[B84] Holý A. (1993). Syntheses of Enantiomeric N-(3-hydroxy-2-phosphonomethoxypropyl) Derivatives of Purine and Pyrimidine Bases. Collect. Czech Chem. Commun. 58 (3), 649–674. 10.1135/cccc19930649

[B85] Holý A. (2005). “Synthesis of Acyclic Nucleoside Phosphonates,” in Current Protocols In Nucleic Acid Chemistry”, 2005, Oct., Chapter 14, Unit 14.2, Hoboken, NJ, United States: John Wiley & Sons. 10.1002/0471142700.nc1402s22 18428938

[B86] Holý A. Votruba I. Masojídková M. Andrei G. Snoeck R. Naesens L. (2002). 6-[2-(Phosphonomethoxy)alkoxy]pyrimidines with Antiviral Activity. J. Med. Chem. 45 (9), 1918–1929. 10.1021/jm011095y 11960502

[B87] Holý A. Votruba I. Tloušťová E. Masojídková M. (2001). Synthesis and Cytostatic Activity of N-[2-(phosphonomethoxy)alkyl] Derivatives of N-6 Substituted Adenines, 2,6- Diaminopurines and Related Compounds. Collect. Czech Chem. Commun. 66 (10), 1545–1592. 10.1135/cccc20011545

[B88] Hostetler K. Y. Aldern K. A. Wan W. B. Ciesla S. L. Beadle J. R. (2006). Alkoxyalkylesters of (S)-HPMPA Are Potent Inhibitors of HIV-1 Replication, *In Vitro* . Antimicrob.Agents Chemother. 50, 2857–2859. 16870786 10.1128/AAC.01223-05 PMC1538655

[B89] Hostetler K. Y. (2009). Alkoxyalkyl Prodrugs of Acyclic Nucleoside Phosphonates Enhance Oral Antiviral Activity and Reduce Toxicity: Current State of the Art. Antivir. Res. 82 (2), A84–A98. 10.1016/j.antiviral.2009.01.005 19425198 PMC2768545

[B90] Izzedine H. Hulot J. S. Launay-Vacher V. Marcellini P. Hadziyannis S. J. Currie G. (2004). Renal Safety of Adefovir Dipivoxil in Patients with Chronic Hepatitis B: Two Double-Blind, Randomized, Placebo-Controlled Studies. Kidney Int. 66 (3), 1153–1158. 10.1111/j.1523-1755.2004.00866.x 15327411

[B91] Jackson P. F. Cole D. C. Slusher B. S. Stetz S. L. Ross L. E. Donzanti B. A. (1996). Design, Synthesis, and Biological Activity of a Potent Inhibitor of the Neuropeptidase N-Acetylated Alpha-Linked Acidic Dipeptidase. J. Med. Chem. 39 (2), 619–622. 10.1021/jm950801q 8558536

[B92] Jackson P. F. Slusher B. S. (2001). Design of NAALADase Inhibitors: A Novel Neuroprotective Strategy. Curr. Med. Chem. 8 (8), 949–957. 10.2174/0929867013372797 11375762

[B93] Jaffe I. A. (1986). Adverse Effects Profile of Sulfhydryl Compounds in Man. Am. J. Med. 80 (3), 471–476. 10.1016/0002-9343(86)90722-9 2937293

[B94] Janczura K. J. Olszewski R. T. Bzdega T. Bacich D. J. Heston W. D. Neale J. H. (2013). NAAG Peptidase Inhibitors and Deletion of NAAG Peptidase Gene Enhance Memory in Novel Object Recognition Test. Eur. J. Pharmacol. 701 (1-3), 27–32. 10.1016/j.ejphar.2012.11.027 23200894 PMC3594592

[B95] Jansa P. Baszczyňski O. Dračínský M. Votruba I. Zídek Z. Bahador G. (2011). A Novel and Efficient One-Pot Synthesis of Symmetrical Diamide (Bis-amidate) Prodrugs of Acyclic Nucleoside Phosphonates and Evaluation of Their Biological Activities. Eur. J. Med. Chem. 46 (9), 3748–3754. 10.1016/j.ejmech.2011.05.040 21664011

[B96] Jindřich J. Holý A. Dvořáková H. (1993). Synthesis of N-(3-fluoro-2-phosphonomethoxypropyl) (FPMP) Derivatives of Heterocyclic Bases. Collect. Czech Chem. Commun. 58 (7), 1645–1667. 10.1135/cccc19931645

[B97] Jones D., J. O´Leary E., M. O´Sullivan T., P. (2019). An Improved Synthesis of Adefovir and Related Analogues. Beil. J. Org. Chem. 15, 801–810. 10.3762/bjoc.15.77 PMC6444443 30992729

[B98] Jones W. Griffiths K. Barata P. C. Paller C. J. (2020). PSMA Theranostics: Review of the Current Status of PSMA-Targeted Imaging and Radioligand Therapy. Cancers 12 (6), 1367. 10.3390/cancers12061367 PMC7352725 32466595

[B99] Jung Y. W. Kim M. Kim B. K. Park J. Y. Kim D. Ahn S. H. (2020). Influence of Besifovir Dipivoxil Maleate Combined with L-Carnitine on Hepatic Steatosis in Patients with Chronic Hepatitis B. J. Korean Med. Sci. 35 (17), e104. 10.3346/jkms.2020.35.e104 32356416 PMC7200179

[B100] Kahn J. Lagakos S. Wulfsohn M. Cherng D. Miller M. Cherrington J. (1999). Efficacy and Safety of Adefovir Dipivoxil with Antiretroviral Therapy: a Randomized Controlled Trial. JAMA- J. Am. Med. Assoc. 282 (24), 2305–2312. 10.1001/jama.282.24.2305 10612317

[B101] Kaittanis C. Andreou C. Hieronymus H. Mao N. Foss C. A. Eiber M. (2018). Prostate-specific Membrane Antigen Cleavage of Vitamin B9 Stimulates Oncogenic Signaling through Metabotropic Glutamate Receptors. J. Exp. Med. 215 (1), 159–175. 10.1084/jem.20171052 29141866 PMC5748857

[B102] Kalčic F. Frydrych J. Doležalová E. Slapničková M. Pachl P. Poštová Slavětínská L. (2021). C1 '-Branched Acyclic Nucleoside Phosphonates Mimicking Adenosine Monophosphate: Potent Inhibitors of Trypanosoma Brucei Adenine Phosphoribosyltransferase. Eur. J. Med. Chem. 225, 113798. 10.1016/j.ejmech.2021.113798 34482272

[B103] Kalčic F. Zgarbová M. Hodek J. Chalupský K. Dračínský M. Dvořáková A. (2021). Discovery of Modified Amidate (ProTide) Prodrugs of Tenofovir with Enhanced Antiviral Properties. J. Med. Chem. 64 (22), 16425–16449. 10.1021/acs.jmedchem.1c01444 34713696

[B104] Kaminsky R. Schmid C. Grether Y. Holý A. De Clercq E. Naesens L. (1996). (S)-9-(3-hydroxy-2-phosphonylmethoxypropyl)adenine [(S)-HPMPA]: A Purine Analoguewith Trypanocidal Activity *In Vitro* and *In Vivo* . Trop. Med. Int. Health 1 (2), 255–263. 10.1111/j.1365-3156.1996.tb00036.x 8665394

[B105] Kaminsky R. Zweygarth E. De Clercq E. (1994). Antitrypanosomal Activity of Phosphonomethoxyalkylpurines. J. Parasitol. 80 (6), 1026–1030. 10.2307/3283453 7799144

[B106] Keough D. T. Hocková D. Rejman D. Špaček P. Vrbková S. Krečmerová M. (2013a). Inhibition of the Escherichia coli 6-Oxopurine Phosphoribosyltransferases by Nucleoside Phosphonates: Potential for New Antibacterial Agents. J. Med. Chem. 56 (17), 6967–6984. 10.1021/jm400779n 23927482

[B107] Keough D. T. Rejman D. Pohl R. Zborníková E. Hocková D. Croll T. (2018). Design of Plasmodium Vivax Hypoxanthine-Guanine Phosphoribosyltransferase Inhibitors as Potential Antimalarial Therapeutics. ACS Chem. Biol. 13 (1), 82–90. 10.1021/acschembio.7b00916 29161011

[B108] Keough D. T. Špaček P. Hocková D. Tichý T. Vrbková S. Slavětínská L. (2013b). Acyclic Nucleoside Phosphonates Containing a Second Phosphonate Group Are Potent Inhibitors of 6-Oxopurine Phosphoribosyltransferases and Have Antimalarial Activity. J. Med. Chem. 56 (6), 2513–2526. 10.1021/jm301893b 23448281

[B109] Kern E. R. Hartline C. Harden E. Keith K. Rodriguez N. Beadle J. R. (2002). Enhanced Inhibition of Orthopoxvirus Replication *In Vitro* by Alkoxyalkyl Esters of Cidofovir and Cyclic Cidofovir. Antimicrob. Agents Chemother. 46 (4), 991–995. 10.1128/AAC.46.4.991-995.2002 11897580 PMC127114

[B110] Kim D. H. Sung D. H. Min Y. K. (2013). Hypophosphatemic Osteomalacia Induced by Low-Dose Adefovir Therapy: Focus on Manifestations in the Skeletal System and Literature Review. J. Bone Min. Metab. 31 (2), 240–246. 10.1007/s00774-012-0384-y 22976054

[B111] Klejch T. Keough D. T. Chavchich M. Travis J. Skácel J. Pohl R. (2019). Sulfide, Sulfoxide and Sulfone Bridged Acyclic Nucleoside Phosphonates as Inhibitors of the Plasmodium Falciparum and Human 6-oxopurine Phosphoribosyltransferases: Synthesis Andevaluation. Eur. J. Med. Chem. 183, 111667. 10.1016/j.ejmech.2019.111667 31536893

[B112] Knejzlík Z. Herkommerová K. Hocková D. Pichová I. (2020). Hypoxanthine-Guanine Phosphoribosyltransferase Is Dispensable for Mycobacterium Smegmatis Viability. J. Bacteriol. 202 (5), e00710–19. 10.1128/JB.00710-19 31818925 PMC7015712

[B113] Kramata P. Downey K. M. Paborsky L. R. (1998). Incorporation and Excision of 9-(2- Phosphonylmethoxyethyl)guanine (PMEG) by DNA Polymerase Delta and Epsilon *In Vitro* . J. Biol. Chem. 273 (34), 21966–21971. 10.1074/jbc.273.34.21966 9705337

[B114] Kramata P. Votruba I. Otová B. Holý A. (1996). Different Inhibitory Potencies of Acyclic Phosphonomethoxyalkyl Nucleotide Analogs toward DNA Polymerases α, δ, and ε. Mol. Pharmacol. 49 (6), 1005 8649338

[B115] Kratochwil C. Giesel F. L. Leotta K. Eder M. Hoppe-Tich T. Youssoufian H. (2015). PMPA for Nephroprotection in PSMA-Targeted Radionuclide Therapy of Prostate Cancer. J. Nucl. Med. 56 (2), 293–298. 10.2967/jnumed.114.147181 25613537

[B116] Krečmerová M. (2017). Amino Acid Esters Prodrugs of Nucleoside and Nucleotide Antivirals. Mini Rev. Med. Chem. 17 (10), 818–833. 10.2174/1389557517666170216151601 28215138

[B117] Krečmerová M. Dračínský M. Hocková D. Holý A. Keough D. T. Guddat L. W. (2012). Synthesis of Purine N^9^-[2-Hydroxy-3-O-(phosphonomethoxy)propyl] Derivatives and Their Side-Chain Modified Analogues as Potential Antimalarial Agents. Bioorg. Med. Chem. 20 (3), 1222–1230. 10.1016/j.bmc.2011.12.034 22249123

[B118] Krečmerová M. Dračínský M. Snoeck R. Balzarini J. Pomeisl K. Andrei G. (2017). New Prodrugs of Two Pyrimidine Acyclic Nucleoside Phosphonates: Synthesis and Antiviral Activity. Bioorg. Med. Chem. 25 (17), 4637–4648. 10.1016/j.bmc.2017.06.046 28757102 PMC7126465

[B119] Krečmerová M. Holý A. Andrei G. Pomeisl K. Tichý T. Břehová P. (2010). Synthesis of Ester Prodrugs of 9-(S)-[3-hydroxy-2-(phosphonomethoxy)propyl]-2,6-diaminopurine (HPMPDAP) as Anti-poxvirus Agents. J. Med. Chem. 53 (19), 6825–6837. 10.1021/jm901828c 20809641

[B120] Krečmerová M. Holý A. Pískala A. Masojídková M. Andrei G. Naesens L. (2007a). Antiviral Activity of Triazine Analogues of 1-(S)-[3- Hydroxy-2-(phosphonomethoxy)propyl]cytosine (Cidofovir) and Related Compounds. J. Med. Chem. 50 (5), 1069–1077. 10.1021/jm061281+ 17298047

[B121] Krečmerová M. Holý A. Pohl R. Masojidková M. Andrei G. Naesens L. (2007b). Ester Prodrugs of Cyclic 1-(S)-[3-hydroxy-2-(phosphonomethoxy)propyl]-5-azacytosine: Synthesis and Antiviral Activity. J. Med. Chem. 50 (23), 5765–5772. 10.1021/jm0707166 17948980

[B122] Krečmerová M. Jansa P. Dračínský M. Sázelová P. Kašička V. Neyts J. (2013). 9-[2-(R)-(Phosphonomethoxy)propyl]-2,6-diaminopurine (R)-PMPDAP and its Prodrugs: Optimized Preparation, Including Identification of By-Products Formed, and Antiviral Evaluation *In Vitro* . Bioorg. Med. Chem. 21 (5), 1199–1208. 10.1016/j.bmc.2012.12.044 23375089 PMC7127208

[B123] Kreider J. W. Balogh K. Olson R. O. Martin J. C. (1990). Treatment of Latent Rabbit and Human Papillomavirus Infections with 9-(2-phosphonylmethoxy)ethylguanine (PMEG). Antivir. Res. 14 (1), 51–58. 10.1016/0166-3542(90)90065-F 1964372

[B124] Krylov I. S. Kashemirov B. A. Hilfinger J. M. McKenna C. E. (2013). Evolution of an Amino Acid Based Prodrug Approach: Stay Tuned. Mol. Pharm. 10 (2), 445–458. 10.1021/mp300663j 23339402 PMC3788118

[B125] Lacy S. A. Hitchcock M. J. Lee W. A. Tellier P. Cundy K. C. (1998). Effect of Oral Probenecid Coadministration on the Chronic Toxicity and Pharmacokinetics of Intravenous Cidofovir in Cynomolgus Monkeys. Toxicol. Sci. 44 (2), 97–106. 10.1006/toxs.1998.2481 9742650

[B126] Lee W. A. He G.-X. Eisenberg E. Cihlar T. Swaminathan S. Mulato A. (2005). Selective Intracellular Activation of a Novel Prodrug of the Human Immunodeficiency Virus Reverse Transcriptase Inhibitor Tenofovir Leads to Preferential Distribution and Accumulation in Lymphatic Tissue. Antimicrob. Agents Chemother. 49 (5), 1898−1906. 10.1128/AAC.49.5.1898-1906.2005 15855512 PMC1087627

[B127] Liu D. Shi B. Wang F. Yu R. Hung C. (2013). Tenofovir Alafenamide hemifumarate. U.S. Patent No WO2013025788 A1. Foster City, CA, United States: Gilead Sciences, Inc. Publication date, 21.02. 2013.

[B128] Luo M. Groaz E. Andrei G. Snoeck R. Kalkeri R. Ptak R. G. (2017). Expanding the Antiviral Spectrum of 3-Fluoro-2-(phosphonomethoxy)propyl Acyclic Nucleoside Phosphonates: Diamyl Aspartate Amidate Prodrugs. J. Med. Chem. 60 (14), 6220–6238. 10.1021/acs.jmedchem.7b00416 28682067

[B129] Luo M. Groaz E. De Jonghe S. Snoeck R. Andrei G. Herdewijn P. (2018). Amidate Prodrugs of Cyclic 9-(S)-[3-Hydroxy-2(phosphonomethoxy)propyl]adenine with Potent Anti-herpesvirus Activity. ACS Med. Chem. Lett. 9 (4), 381–385. 10.1021/acsmedchemlett.8b00079 29670705 PMC5900341

[B130] Luszczki J. J. Mohamed M. Czuczwar S. J. (2006). 2-phosphonomethyl-pentanedioic Acid (Glutamate Carboxypeptidase II Inhibitor) Increases Threshold for Electroconvulsionsions and Enhances the Antiseizure Action of Valproate against Maximal Electroshock-Induced Seizures in Mice. Eur. J. Pharmacol. 531 (1-3), 66–73. 10.1016/j.ejphar.2005.11.045 16403497

[B131] Lyseng-Williamson K. A. Reynolds N. A. Plosker G. L. (2005). Tenofovir Disoproxil Fumarate - A Review of its Use in the Management of HIV Infection. Drugs 65 (3), 413–432. 10.2165/00003495-200565030-00006 15669881

[B132] Majer P. Jackson P. F. Delahanty G. Grella B. S. Ko Y. S. Li W. (2003). Synthesis and Biological Evaluation of Thiol-Based Inhibitors of Glutamate Carboxypeptidase II: Discovery of an Orally Active GCP II Inhibitor. J. Med. Chem. 46 (10), 1989–1996. 10.1021/jm020515w 12723961

[B133] Majer P. Jančařík A. Krečmerová M. Tichý T. Tenora L. Wozniak K. (2016). Discovery of Orally Available Prodrugs of the Glutamate Carboxypeptidase II (GCP II) Inhibitor 2-phosphonomethyl Pentanedioic Acid (2-PMPA). J. Med. Chem. 59 (6), 2810–2819. 10.1021/acs.jmedchem.6b00062 26930119

[B134] Marrazzo J. M. Ramjee G. Richardson B. A. Gomez K. Mgodi N. M. Nair G. (2015). Tenofovir-based Preexposure Prophylaxis for HIV Infection Among African Women. N. Engl. J. Med. 372 (6), 509–518. 10.1056/NEJMoa1402269 25651245 PMC4341965

[B135] Marty F. M. Winston D. J. Chemaly R. F. Mullane K. M. Shore T. B. Papanicolaou G. A. (2019). A Randomized, Double-Blind, Placebo-Controlled Phase 3 Trial of Oral Brincidofovir for Cytomegalovirus Prophylaxis in Allogeneic Hematopoietic Cell Transplantation. Biol. Blood Marrow Transpl. 25 (2), 369–381. 10.1016/j.bbmt.2018.09.038 PMC8196624 30292744

[B136] McKinzie D. L. Li T. K. McBride W. J. Slusher B. S. (2000). NAALADase Inhibition Reduces Alcohol Consumption in the Alcohol‐preferring (P) Line of Rats. Addict. Biol. 5 (4), 411–416. 10.1111/j.1369-1600.2000.tb00209.x 20575858

[B137] Meier C. Balzarini J. (2006). Application of the Sal-Prodrug Approach for Improving the Biological Potential of Phosphorylated Biomolecules. Antivir. Res. 71 (2-3), 282–292. 10.1016/j.antiviral.2006.04.011 16735066

[B138] Meier C. (2006). CycloSal Phosphates as Chemical Trojan Horses for Intracellular Nucleotide and Glycosylmonophosphate Delivery - Chemistry Meets Biology. Eur. J. Org. Chem. (5), 1081–1102. 10.1002/ejoc.200500671

[B139] Meier C. Görbig U. Müller C. Balzarini C. (2005). cycloSal-PMEA and cycloAmb-PMEA: Potentially New Phosphonate Prodrugs Based on the cycloSal-Pronucleotide Approach. J. Med. Chem. 48 (25), 8079–8086. 10.1021/jm050641a 16335932

[B140] Murata K. Tsukuda S. Suizu F. Kimura A. Sugiyama S. Watashi K. (2020). Immunomodulatory Mechanism of Acyclic Nucleoside Phosphates in Treatment of Hepatitis B Virus Infection. Hepatology 71 (5), 1533–1545. 10.1002/hep.30956 31529730

[B141] Nachega J. B. Uthman O. A. Mofenson L. M. Anderson J. R. Kanters S. Renaud F. (2017). Safety of Tenofovir Disoproxil Fumarate-Based Antiretroviral Therapy Regimens in Pregnancy for HIV-Infected Women and Their Infants: A Systematic Review and Meta Analysis. J. Acquir Immune Defic. Syndr. 76 (1), 1–12. 10.1097/qai.0000000000001359 28291053 PMC5553236

[B142] Naesens L. Andrei G. Votruba I. Krečmerová M. Holý A. Neyts J. (2008). Intracellular Metabolism of the New Antiviral Compound, 1-(S)-[3-hydroxy-2--(phosphonomethoxy)propyl]-5-azacytosine. Biochem. Pharmacol. 76 (8), 997–1005. 10.1016/j.bcp.2008.08.009 18773877

[B143] Naesens L. Balzarini J. De Clercq E. (1994). Therapeutic Potential of PMEA as an Antiviral Drug. Rev. Med. Virol. 4 (3), 147–159. 10.1002/rmv.1980040302

[B144] Najjar A. Karaman R. (2019). The Prodrug Approach in the Era of Drug Design. Expert Opin. Drug Del 16 (1), 1–5. 10.1080/17425247.2019.1553954 30558447

[B145] Neale J. H. Yamamoto T. (2020). N-acetylaspartylglutamate (NAAG) and Glutamate Carboxypeptidase II: An Abundant Peptide Neurotransmitter-Enzyme System with Multiple Clinical Applications. Prog. Neurobiol. 184, 101722. 10.1016/j.pneurobio.2019.101722 31730793

[B146] Neant N. Klifa R. Bouazza N. Moshous D. Neven B. Leruez-Ville B. (2018). Model of Population Pharmacokinetics of Cidofovir in Immunocompromised Children with Cytomegalovirus and Adenovirus Infections. J. Antimicrob. Chemother. 73 (9), 2422–2429. 10.1093/jac/dky192 29860512

[B147] Nedelcovych M. Dash R. P. Tenora L. Zimmermann S. C. Gadiano A. J. Garrett C. (2017). Enhanced Brain Delivery of 2-(Phosphonomethyl)pentanedioic Acid Following Intranasal Administration of its γ-Substituted Ester Prodrugs. Mol. Pharm. 14 (10), 3248–3257. 10.1021/acs.molpharmaceut.7b00231 28763226 PMC5795618

[B148] Neofytos D. Ojha A. Mookerjee B. Wagner J. Filicko J. Ferber A. (2007). Treatment of Adenovirus Disease in Stem Cell Transplant Recipients with Cidofovir. Biol. Blood Marrow Transpl. 13 (1), 74–81. 10.1016/j.bbmt.2006.08.040 17222755

[B149] Nguyen T. Kirsch B. J. Asaka R. Nabi K. Quinones A. Tan J. (2019). Uncovering the Role of N-Acetyl-Aspartyl-Glutamate as a Glutamate Reservoir in Cancer. Cell Rep. 27 (2), 491–501. 10.1016/j.celrep.2019.03.036 30970252 PMC6472703

[B150] Nonaka T. Yamada T. Ishimura T. Zuo D. Y. Moffett J. R. Neale J. H. (2017). A Role for the Locus Coeruleus in the Analgesic Efficacy of N-Acetylaspartylglutamate Peptidase (GCPII) Inhibitors ZJ43 and 2-PMPA. Mol. Pain 13, 1–13. 10.1177/1744806917697008 PMC5407666 28326936

[B151] Oh C. H. Hong J. H. (2008). Design, Synthesis and Anti-HIV Activity of Homologous PMEA Derivatives. Nucleosides Nucleotides Nucleic Acids 27 (2), 186–195. 10.1080/15257770701795953 18205072

[B152] Olszewski R. T. Janczura K. J. Ball S. R. Madore J. C. Lavin K. M. Lee J. C. (2012). NAAG Peptidase Inhibitors Block Cognitive Deficit Induced by MK-801 and Motor Activation Induced by D-Amphetamine in Animal Models of Schizophrenia. TranslPsychiatry 2, e145. 10.1038/tp.2012.68 PMC3410622 22850437

[B153] Olszewski R. T. Janczura K. J. Bzdega T. Der E. K. Venzor F. O´Rourke B. (2017). NAAG Peptidase Inhibitors Act via mGluR3: Animal Models of Memory, Alzheimer's, and Ethanol Intoxication. Neurochem. Res. 42 (9), 2646–2657. 10.1007/s11064-017-2181-4 28285415 PMC5603630

[B154] Park S. Kim W. I. Cho D.-H. Kim Y. J. Kim H. S. Kim J. H. (2018). Adefovir-induced Fanconi Syndrome Associated with Osteomalacia. Clin. Mol. Hepatol. 24 (3), 339–344. 10.3350/cmh.2017.0009 28859264

[B155] Peters D. E. Norris L. D. Slusher B. S. (2019). Spontaneous Loss-Of-Function Dock2 Mutation Alters Murine Colitis Sensitivity and Is a Confounding Variable in Inflammatory Bowel Disease Research. Crohn's Colitis 360, otz030. 10.1093/crocol/otz030 PMC11610769 39624652

[B156] Peters D. Norris L. Tenora L. Šnajdr I. Zhu X. Sakamoto S. (2022). Discovery of IBD3540: A Novel Gut-Restricted Glutamate Carboxypeptidase II Inhibitor with Oral Activity in Mouse Colitis Models. Inflam. Bowel Dis. 28 (Suppl. 1), S4. 10.1093/ibd/izac015.007

[B157] Peterson L. W. Kim J. S. Kijek P. Mitchell S. Hilfinger J. M. Breitenbach J. M. (2011). Synthesis, Transport and Antiviral Activity of Ala-Ser and Val-Ser Prodrugs of Cidofovir. Bioorg. Med. Chem. Lett. 21 (13), 4045–4049. 10.1016/j.bmcl.2011.04.126 21641218 PMC3115518

[B158] Pisarev V. M. Lee S.-H. Connelly M. C. Fridland A. (1997). Intracellular Metabolism and Action of Acyclic Nucleoside Phosphonates on DNA Replication. Mol. Pharmacol. 52 (1), 63–68. 10.1124/mol.52.1.63 9224813

[B159] Plosker G. L. Noble S. (1999). Cidofovir - A Review of its Use in Cytomegalovirus Retinitis in Patients with AIDS. Drugs 58 (2), 325–345. 10.2165/00003495-199958020-00015 10473024

[B160] Pomeisl K. Pohl R. Holý A. Votruba I. (2005). Simple Transformation of Thymine 1-[3-hydroxy-2-(phosphonomethoxy)propyl] Derivatives to Their 1-[3-fluoro-2-(phosphonomethoxy)propyl] Counterparts. Collect. Czech Chem. Commun. 70 (9), 1465–1481. 10.1135/cccc20051465

[B161] Pomeislová A. Otmar M. Rubešová P. Benýšek J. Matoušová M. Mertlíková-Kaiserová H. (2021). 1,2,4-Thiadiazole Acyclic Nucleoside Phosphonates as Inhibitors of Cysteine Dependent Enzymes Cathepsin K and GSK-3β. Bioorg. Med. Chem. 32, 115998. 10.1016/j.bmc.2021.115998 33440320

[B162] Pradere U. Garnier-Amblard E. C. Coats S. J. Amblard F. Schinazi R. F. (2014). Synthesis of Nucleoside Phosphate and Phosphonate Prodrugs. Chem. Rev. 114 (18), 9154–9218. 10.1021/cr5002035 25144792 PMC4173794

[B163] Rahn K. A. Watkins C. C. Alt J. Rais R. Stathis M. Grishkan I. (2012). Inhibition of Glutamate Carboxypeptidase II (GCPII) Activity as a Treatment for Cognitive Impairment in Multiple Sclerosis. Proc. Natl. Acad. Sci. U. S. A. 109 (49), 20101–20106. 10.1073/pnas.1209934109 23169655 PMC3523869

[B164] Rais R. Jiang W. W. Zhai H. H. Wozniak K. M. Stathis M. Hollinger K. R. (2016). FOLH1/GCPII Is Elevated in IBD Patients, and its Inhibition Ameliorates Murine IBD Abnormalities. JCI Insight 1 (12), e88634. 10.1172/jci.insight.88634 27536732 PMC4985244

[B165] Rais R. Wozniak K. Wu Y. Niwa M. Stathis M. Alt J. (2015). Selective CNS Uptake of the GCP-II Inhibitor 2-PMPA Following Intranasal Administration. Plos One 10 (7), e0131861. 10.1371/journal.pone.0131861 26151906 PMC4494705

[B166] Ramanathan S. (2013). Combination Therapy Comprising Tenofovir Alafenamide Hemifumarate and Cobicistat for Use in the Treatment of Viral Infections. Foster City, CA, United States: Gilead Sciences, Inc. Publication date. U.S. Patent No. WO/2013/116730-08-08.

[B167] Rautio J. Kumpulainen H. Heimbach T. Oliyai R. Oh D. Järvinen T. (2008). Prodrugs: Design and Clinical Applications. Nat. Rev. Drug Discov. 7 (3), 255–270. 10.1038/nrd2468 18219308

[B168] Reddy K. R. Matelich M. C. Ugarkar B. G. Gomez-Galeno J. E. DaRe J. Ollis K. (2008). Pradefovir: A Prodrug that Targets Adefovir to the Liver for the Treatment of Hepatitis B. J. Med. Chem. 51 (3), 666–676. 10.1021/jm7012216 18173234

[B169] Reiser H. Wang J. Y. Chong L. Watkins W. J. Ray A. S. Shibata R. (2008). GS-9219 - A Novel Acyclic Nucleotide Analogue with Potent Antineoplastic Activity in Dogs with Spontaneous Non–hodgkin's Lymphoma. Clin. Cancer Res. 14 (9), 2824–2832. 10.1158/1078-0432.CCR-07-2061 18451250

[B170] Reymen D. Naesens L. Balzarini J. Holý A. Dvořáková H. De Clercq E. (1995). Antiviral Activity of Selected Acyclic Nucleoside Analogues against Human Herpesvirus 6. Antivir. Res. 28 (4), 343–357. 10.1016/0166-3542(95)00058-5 8669893

[B171] Roels S. Van der Heyden S. Neyts J. Krečmerová M. Koenen F. Cay A. B. (2013). Safety Assessment in Pigs of an Experimental Molecule with *In-Vitro* Antiviral Activity against African Swine Fever. J. Comp. Pathol. 148 (1), 94. 10.1016/j.jcpa.2012.11.194

[B172] Rose W. C. Crosswell A. R. Bronson J. J. Martin J. C. (1990). *In Vivo* tumor Activity of 9-[(2-Phosphonylmethoxy)ethyl]-Guanine (PMEG) and Related Phosphonate Nucleotide Analogs. J. Natl. Cancer Inst. 82 (6), 510–512. 10.1093/jnci/82.6.510 2313724

[B173] Ruiz J. Beadle J. R. Buller R. M. Schreiwer J. Prichard M. N. Keith K. A. (2011). Synthesis, Metabolic Stability and Antiviral Evaluation of Various Alkoxyalkyl Esters of Cidofovir and 9-(S)-[3-hydroxy-2-(phosphonomethoxy)propyl]adenine. Bioorg. Med. Chem. 19 (9), 2950–2958. 10.1016/j.bmc.2011.03.034 21493074 PMC3104040

[B174] Schinkmanová M. Votruba I. Holý A. (2006). N-6-Methyl-AMP Aminohydrolase Activates N-6-Substituted Purine Acyclic Nucleoside Phosphonates. Biochem. Pharmacol. 71 (9), 1370–1376. 10.1016/j.bcp.2006.01.013 16513094

[B175] Schinkmanová M. Votruba I. Shibata R. Han B. Liu X. H. Cihlář T. (2008). Human N-6-Methyl-AMP/damp Aminohydrolase (Abacavir 5 '-monophosphate Deaminase) Is Capable of Metabolizing N-6-Substituted Purine Acyclic Nucleoside Phosphonates. Collect. Czech. Chem. Commun. 73 (2), 275–291. 10.1135/cccc20080275

[B176] Seley-Radtke K. L. Yates M. K. (2018). The Evolution of Nucleoside Analogue Antivirals: A Review for Chemists and Non-chemists. Part 1: Early Structural Modifications to the Nucleoside Scaffold. Antivir. Res. 154, 66–86. 10.1016/j.antiviral.2018.04.004 29649496 PMC6396324

[B177] Shaw J. P. Louie M. S. Krishnamurty V. V. Arimilli M. N. Jones R. J. Bidgood A. M. (1997). Pharmacokinetics and Metabolism of Selected Prodrugs of PMEA in Rats. Drug Metab. Dispos. 25 (3), 362 9172955

[B178] Siberry G. K. Williams P. L. Mendez H. Seage G. R. III Jacobson D. L. Hazra R. (2012). Safety of Tenofovir Use during Pregnancy: Early Growth Outcomes in HIV-Exposed Uninfected Infants. AIDS 26 (9), 1151–1159. 10.1097/QAD.0b013e328352d135 22382151 PMC3476702

[B179] Slusher B. S. Robinson M. B. Tsai G. C. Simmons M. L. Richards S. S. Coyle J. T. (1990). Rat-brain N-Acetylated Alpha-Linked Acidic Dipeptidase Activity – Purification and Immunological Characterization. J. Biol. Chem. 265 (34), 21297–21301. 10.1016/s0021-9258(17)45359-2 2250024

[B180] Slusher B. S. Vornov J. J. Thomas A. G. Hurn P. D. Harukuni I. Bhardwaj A. (1999). Selective Inhibition of NAALADase, Which Converts NAAG to Glutamate, Reduces Ischemic Brain Injury. Nat. Med. 5 (12), 1396–1402. 10.1038/70971 10581082

[B181] Šmídková M. Dvořáková A. Tloušťová E. Česnek M. Janeba Z. Mertlíková-Kaiserová H. (2014). Amidate Prodrugs of 9-[2-(phosphonomethoxy)ethyl]adenine as Inhibitors of Adenylate Cyclase Toxin from Bordetella Pertusis. Antimicrob. Agents Chemother. 58 (2), 664–671. 10.1128/AAC.01685-13 24145524 PMC3910841

[B182] Smith W. R. Neill J. Cushman W. C. Butkus D. E. (1989). Captopril-Associated Acute Interstitial Nephritis. Am. J. Nephrol. 9 (3), 230–235. 10.1159/000167970 2527007

[B183] Srinivas R. V. Fridland A. (1998). Antiviral Activities of 9-R-2-Phosphonomethoxypropyl Adenine (PMPA) and Bis(isopropyloxymethylcarbonyl) PMPA against Various Drug-Resistant Human Immunodeficiency Virus Strains. Antimicrob. Agents Chemother. 42 (6), 1484–1487. 10.1128/AAC.42.6.1484 9624498 PMC105626

[B184] Starrett J. E. Jr. Tortolani D. R. Hitchcock M. J. Martin J. C. Mansuri M. M. (1992). Synthesis and in Evaluation of a Phosphonate Prodrug: Bis(pivaloyloxymethyl) 9-(2- Phosphonylmethoxyethyl)adenine. Antivir. Res. 19 (3), 267–273. 10.1016/0166-3542(92)90084-I 1332606

[B185] Stoermer D. Liu Q. Hall M. R. Flanary J. M. Thomas A. G. Rojas C. (2003). Synthesis and Biological Evaluation of Hydroxamate-Based Inhibitors of Glutamate Carboxypeptidase II. Bioorg. Med. Chem. Lett. 13 (13), 2097–2100. 10.1016/S0960-894X(03)00407-4 12798312

[B186] Suo Z. Johnson K. A. (1998). Selective Inhibition of HIV-1 Reverse Transcriptase by an Antiviral Inhibitor, (R)-9-(2-Phosphonylmethoxypropyl) Adenine. J. Biol. Chem. 273 (42), 27250–27258. 10.1074/jbc.273.42.27250 9765248

[B187] Taffin E. Paepe D. Goris N. Auwerx J. Debille M. Neyts J. (2015). Antiviral Treatment of Feline Immunodeficiency Virus-Infected Cats with (R)-9-(2- Phosphonylmethoxypropyl)-2,6-Diaminopurine. L. Feline Med. Surg. 17 (2), 79–86. 10.1177/1098612X14532089 PMC10816418 24782459

[B188] Tallon C. Sharma A. Zhang Z. Thomas A: G. Ng J. Zhu X. L. (2022). Dendrimer-2PMPA Delays Muscle Function Loss and Denervation in a Murine Model of Amyotrophic Lateral Sclerosis. Neurotherapeutics 1, 15. 10.1007/s13311-021-01159-7 PMC9130402 34984651

[B189] Teran D. (2020). Acyclic Nucleoside Phosphonates as Possible Chemotherapeutics against Trypanosoma Brucei. Drug Discov. Today 25 (6), 1043–1053. 10.1016/j.drudis.2020.02.008 32135205

[B190] Tillmann H. L. (2007). Drug Evaluation: Pradefovir, a Liver-Targeted Prodrug of Adefovir against HBV Infection. Curr. Opin. Invest.. Drugs 8 (8), 682. 17668370

[B191] Tillmann H. L. Samuel G. (2019). Current State-Of-The-Art Pharmacotherapy for the Management of Hepatitis B Infection. Expert Opin. Pharmacother. 20 (7), 873–885. 10.1080/14656566.2019.1583744 30857443

[B192] Vahlenkamp T. W. Deronde A. Balzarini J. Naesens L. De Clercq E. Vaneijk M. J. T. (1995). (R)-9-(2-phosphonylmethoxypropyl)-2,6-diaminopurine Is a Potent Inhibitor of Feline Immunodeficiency Virus-Infection. Antimicrob. Agents Chemother. 39 (3), 746–749. 10.1128/AAC.39.3.746 7793884 PMC162616

[B193] Valiaeva N. Prichard M. N. Buller R. M. Beadle J. R. Hartline C. B. Keith K. A. (2009). Antiviral Evaluation of Octadecyloxyethyl Esters of (S)-3-hydroxy-2-(phosphonomethoxy)propyl Nucleosides against Herpesviruses and Orthopoxviruses. Antivir. Res. 84 (3), 254–259. 10.1016/j.antiviral.2009.09.012 19800369 PMC2787864

[B194] Valiaeva N. Wyles D. L. Schooley R. T. Hwu J. B. (2006). Synthesis and Antiviral Evaluation of 9-(S)-[3-alkoxy-2--(phosphonomethoxy)propyl]nucleoside Alkoxyalkyl Esters: Inhibitors of Hepatitis C Virus and HIV-1 Replication. Bioorg. Med. Chem. 19 (15), 4616–4625. 10.1016/j.bmc.2011.06.009 PMC3139790 21719300

[B195] Van Damme L. Corneli A. Ahmed K. Agot K. Lombaard J. Kapiga S. (2012). Preexposure Prophylaxis for HIV Infection Among African Women. N. Engl. J. Med. 367 (5), 411–422. 10.1056/NEJMoa1202614 22784040 PMC3687217

[B196] van der Post J. P. de Visser S. J. de Kam M. L. Woelfler M. Hilt D. C. Vornov J. (2005). The Central Nervous System Effects, Pharmacokinetics and Safety of the NAALADase-Inhibitor GPI 5693. Br. J. Clin. Pharmacol. 60 (2), 128–136. 10.1111/j.1365-2125.2005.02396.x 16042665 PMC1884920

[B197] van Gelder J. Deferme S. Naesens L. De Clercq E. van den Mooter G. Kinget R. (2002). Intestinal Absorption Enhancement of the Ester Prodrug Tenofovir Disoproxil Fumarate through Modulation of the Biochemical Barrier by Defined Ester Mixtures. Drug. Metab. Dispos. 30 (8), 924–930. 10.1124/dmd.30.8.924 12124311

[B198] Vornov J. J. Hollinger K. R. Jackson P. F. Wozniak K. M. Farah M. H. Majer P. (2016). Still NAAG’ing after All These Years: the Continuing Pursuit of GCPII Inhibitors. Adv. Pharmacol. 76, 215–255. 10.1016/bs.apha.2016.01.007 27288079

[B199] Vornov J. J. Wozniak K. M. Wu Y. Rojas C. Rais R. Slusher B. S. (2013). Pharmacokinetics and Pharmacodynamics of the Glutamate Carboxypeptidase II Inhibitor 2-MPPA Show Prolonged Alleviation of Neuropathic Pain through an Indirect Mechanism. J. Pharmacol. Exp. Ther. 346 (3), 406–413. 10.1124/jpet.113.205039 23776202 PMC4186626

[B200] Votruba I. Tryznová J. Břehová P. Tloušťová E. Horská K. Fanfrlík J. (2010). Inhibition of Human Purine Nucleoside Phosphorylase by Tenofovir Phosphate Congeners. Collect. Czech Chem. Commun. 75 (12), 1249–1257. 10.1135/cccc2010094

[B201] Wang L. M. Kourtis A. P. Ellington S. Legardy-Williams J. Bulterys M. (2013). Safety of Tenofovir during Pregnancy for the Mother and Fetus: A Systematic Review. Clin. Infect. Dis. 57 (12), 1773–1781. 10.1093/cid/cit601 24046310

[B202] Wang R. X. Lin L. Y. Zheng Y. Q. Cao P. Yuchi Z. G. Wu H. Y. (2020). Identification of 2-PMPA as a Novel Inhibitor of Cytosolic Carboxypeptidases. Biochem. Biophys. Res. Commun. 533 (4), 1393–1399. 10.1016/j.bbrc.2020.10.029 33092792

[B203] Williams M. Krylov I. S. Zakharova V. M. Serpi M. Peterson L. W. Krečmerová M. (2011). Cyclic and Acyclic Phosphonate Tyrosine Ester Prodrugs of Acyclic Nucleoside Phosphonates.”in Collect. Symp. Ser. Editor Hocek M., 12, 167. Institute of Organic Chemistry and Biochemistry, ASCR, Prague . 10.1135/css201112167

[B204] Wolf D. L. Rodriguez C. A. Mucci M. Ingrosso A. Duncan B. A. Nickens D. J. (2003). Pharmacokinetics and Renal Effects of Cidofovir with a Reduced Dose of Probenecid in HIV-Infected Patients with Cytomegalovirus Retinitis. J. Clin. Pharmacol. 43 (1), 43–51. 10.1177/0091270002239705 12520627

[B205] Wyles D. L. Kaihara K. A. Korba B. E. Schooley R. T. (2009). The Octadecyloxyethyl Ester of (S)-9-[3-Hydroxy-2-(Phosphonomethoxy) Propyl]Adenine Is a Potent and Selective Inhibitor of Hepatitis C Virus Replication in Genotype 1A, 1B, and 2A Replicons. Antimicrob. Agents Chemother. 53 (6), 2660–2662. 10.1128/aac.01546-08 19289518 PMC2687240

[B206] Xi Z. X. Li X. Peng X. Q. Li J. Chun L. Gardner E. L. (2010). Inhibition of NAALADase by 2-PMPA Attenuates Cocaine-Induced Relapse in Rats: a NAAG-mGluR2/3-Mediated Mechanism. J. Neurochem. 112 (2), 564–576. 10.1111/j.1471-4159.2009.06478.x 19895667 PMC2809121

[B207] Yang D. H. Xie Y. J. Zhao N. F. Pan H. Y. Li M. W. Huang H. J. (2015). Tenofovir Disoproxil Fumarate Is Superior to Lamivudine Plus Adefovir in Lamivudine-Resistant Chronic Hepatitis B Patients. World J. Gastroenterol. 21 (9), 2746–2753. 10.3748/wjg.v21.i9.2746 25759545 PMC4351227

[B208] Yim H. J. Kim W. Ahn S. H. Yang J. M. Jang J. Y. Kweon Y. O. (2020). Besifovir Dipivoxil Maleate 144-Week Treatment of Chronic Hepatitis B: An Open-Label Extensional Study of a Phase 3 Trial. Am. J. Gastroenterol. 115 (8), 1217–1225. 10.14309/ajg.0000000000000605 32355123 PMC7402376

[B209] Zakharova V. M. Serpi M. Krylov I. S. Peterson L. W. Breitenbach J. M. Borysko K. Z. (2011). Tyrosine-Based 1-(S)-[3-Hydroxy-2-(phosphonomethoxy)propyl]cytosine and -adenine ((S)-HPMPC and (S)-HPMPA) Prodrugs: Synthesis, Stability, Antiviral Activity, and *In Vivo* Transport Studies. J. Med. Chem. 54 (16), 5680–5693. 10.1021/jm2001426 21812420 PMC3166236

[B210] Zhang H. B. Koumna S. Pouliot F. Beauregard J. M. Kolinsky M. (2021). PSMA Theranostics: Current Landscape and Future Outlook. Cancers 13 (16), 4023. 10.3390/cancers13164023 34439177 PMC8391520

[B211] Zhang H. Wu M. Zhu X. X. Li C. Y. Li X. J. Jin W. L. (2020). Safety, Efficacy, and Pharmacokinetics of Pradefovir for the Treatment of Chronic Hepatitis B Infection. Antivir. Res. 174, 104693. 10.1016/j.antiviral.2019.104693 31838002

[B212] Zhang W. Murakawa Y. Wozniak K. M. Slusher B. Sima A. A. (2006). The Preventive and Therapeutic Effects of GCPII (NAALADase) Inhibition on Painful and Sensory Diabetic Neuropathy. J. Neurol. Sci. 247 (2), 217–223. 10.1016/j.jns.2006.05.052 16780883

[B213] Zhang W. Slusher B. Murakawa Y. Wozniak K. M. Tsukamoto T. Jackson P. (2002). A. GCPII (NAALADase) Inhibition Prevents Long-Term Diabetic Neuropathy in Type 1 Diabetic BB/Wor Rats. J. Neurol. Sci. 194 (1), 21–28. 10.1016/S0022-510X(01)00670-0 11809162

[B214] Zhang Z. Bassam B. Thomas A. G. Williams M. Liu J. H. Nance E. (2016). Maternal Inflammation Leads to Impaired Glutamate Homeostasis and Up-Regulation of Glutamate Carboxypeptidase II in Activated Microglia in the Fetal/newborn Rabbit Brain. Neurobiol. Dis. 94, 116–128. 10.1016/j.nbd.2016.06.010 27326668 PMC5394739

